# Crystal structures of two charge–transfer com­plexes of benzo[1,2-*c*:3,4-*c*′:5,6-*c*′′]tri­thio­phene (*D*_3*h*_-BTT)

**DOI:** 10.1107/S2056989019013161

**Published:** 2019-09-30

**Authors:** Qian Qin, Joel T. Mague, Haley E. Gould, Samuel E. Vasquez, Anthony E. Heyer

**Affiliations:** a Department of Chemistry and Biochemistry, Loyola University, New Orleans, LA, 70118, USA; b Department of Chemistry, Tulane University, New Orleans, LA 70118, USA

**Keywords:** crystal structure, charge–transfer complex, benzotri­thio­phene, C_60_, TCNQ

## Abstract

Benzo[1,2-*c*:3,4-*c*′:5,6-*c*′′]tri­thio­phene (*D*_3*h*_-BTT) is an easily prepared electron donor that readily forms charge–transfer complexes with organic acceptors. We report here two crystal structures of its charge–transfer complexes with 7,7,8,8-tetra­cyano­quinodi­methane (TCNQ) and buckminsterfullerene (C_60_). The *D*_3*h*_-BTT·TCNQ complex, C_12_H_6_S_3_·C_12_H_4_N_4_, crystallizes with mixed layers of donors and acceptors, with an estimated degree of charge transfer at 0.09 *e*. In the *D*_3*h*_-BTT·C_60_·toluene complex, C_12_H_6_S_3_·C_60_·C_7_H_8_, the central ring of BTT is ‘squeezed’ by the C_60_ mol­ecules from both faces. However, the degree of charge transfer is low. The C_60_ unit is disordered over two sites in a 0.766 (3):0.234 (3) ratio and was refined as a two-component inversion twin.

## Abstract 2

Benzo[1,2-*c*:3,4-*c*′:5,6-*c*"]tri­thio­phene (*D*_3*h*_-BTT) is an easily prepared electron donor that readily forms charge–transfer complexes with organic acceptors. We report here two crystal structures of its charge–transfer complexes with 7,7,8,8-tetra­cyano­quinodi­methane (TCNQ) and buckminsterfullerene (C_60_). The *D*_3*h*_-BTT·TCNQ complex crystallizes with mixed layers of donors and acceptors, with an estimated degree of charge transfer at 0.09 *e*. In the *D*_3*h*_-BTT·C_60_·toluene complex, the central ring of BTT is ‘squeezed’ by the C_60_ mol­ecules from both faces. However, the degree of charge transfer is low.

## Chemical context   

Conjugated sulfur-containing aromatic mol­ecules remain the most popular choices for the preparation of organic electronic materials. They are typically electron-rich. Their planar shapes and the large 3*p* orbitals on sulfur atoms allow extensive inter­molecular orbital overlap in the solid state. Both of these features make them very good candidates as donors in binary charge–transfer (CT) complexes (Holiday *et al.*, 2014 ▸). Although CT complexes have long been known, their potential as electronic materials has not been noted until relatively recently (Goetz *et al.*, 2014 ▸). Previous reports indicate that binary charge–transfer (CT) complexes show high conductivity and other promising optoelectronic properties such as ambipolar transport and photoconductivity (Goetz *et al.*, 2014 ▸). We recently prepared and studied the structures and properties of CT complexes using *C_3h_*-symmetric benzotri­thio­phene (*C*_3*h*_-BTT) as the donor (Qin *et al.*, 2017 ▸) with a variety of organic acceptors. The *D*_3*h*_ isomer of benzotri­thio­phene (*D*_3*h*_-BTT) is the most highly symmetric isomer of the BTTs, and all three of its sulfur atoms point away radially from the central ring. It is also one of the most easily prepared BTT isomers (Hart *et al.*, 1978 ▸). The outwardly directed sulfur atoms might maximize inter­molecular S–S contact, a feature that has proven important in promoting high electrical conductivity in some organic conductors (Saito *et al.*, 2011 ▸). Although Hart *et al.* reported that *D*_3*h*_-BTT formed CT complexes with several acceptor mol­ecules including TCNQ, no structural information for any of the CT complexes was provided. In this communication, we report the X-ray crystal structures of the CT complexes *D*_3*h*_-BTT**·**TCNQ and *D*_3*h*_-BTT**·**C_60_·toluene, the latter of which exhibits the second closest pair of bilateral arene–C_60_ contacts.
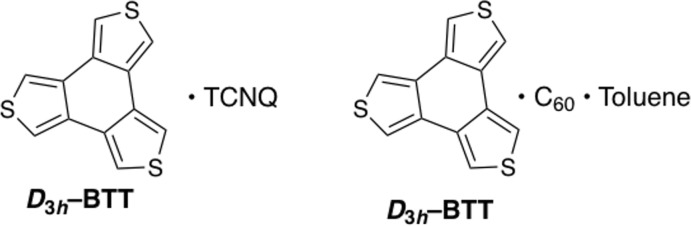


## Structural Commentary   

***D*** **_3_** ***_h_*** **-BTT·TCNQ**

**Figure 1:**
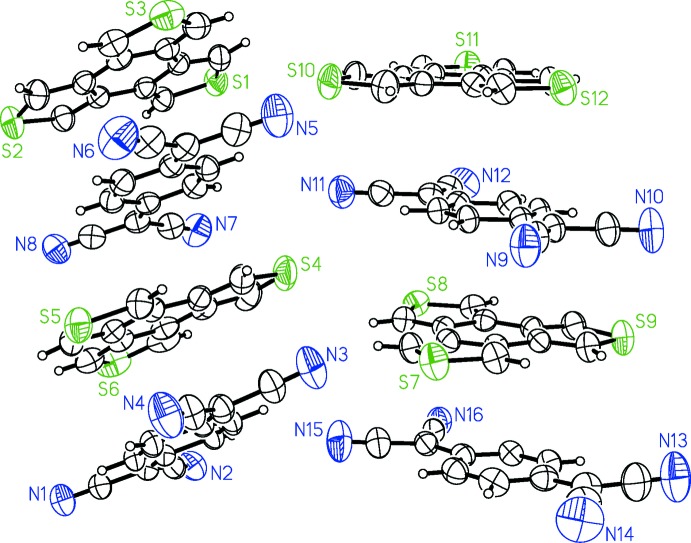
The asymmetric unit of *D*_3h_-BTT·TCNQ. Carbon atom labels have been omitted for clarity.

*D*_3*h*_-BTT forms a 1:1 binary charge–transfer complex with TCNQ, in the space group *P*2_1_/*n*. The asymmetric unit (Fig. 1 ▸) consists of four independent pairs of donor and acceptor mol­ecules arranged in two columns along the *a*-axis of the unit cell. Within the columns, *D*_3*h*_-BTT and TCNQ are stacked pairwise. The π faces of these planar mol­ecules are roughly parallel. The closest donor–acceptor distance is at 3.396 (3) Å (C43⋯C87). The closest contact between the two columns is 3.209 (3) Å (N1⋯S8).

**Figure 2:**
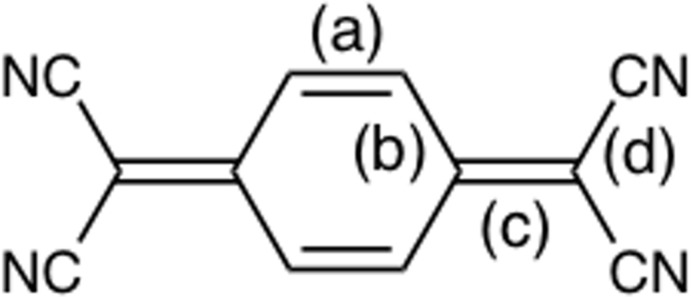
Bonds in TCNQ used to estimate the degree of CT, ρ.

Two methods were used to estimate the extent of charge transfer for the TCNQ complexes. The first is based on bond-distance ratios in the acceptor mol­ecules. The degree of charge transfer is given by ρ = (α_*x*_ – α_0_)/(α_1_ – α_0_), where *α* is the ratio of bond distances *c*/(*b* + *d*) for the indicated bonds in the TCNQ derivative in Fig. 2 ▸. (Kistenmacher *et al.*, 1982 ▸; Sugano *et al.*, 1988 ▸).

The degree of charge transfer based on the bond ratio is 0.09 *e*. This is very close to the degree of charge transfer of *C*_3*h*_-BTT·TCNQ·toluene (0.10 *e*). A second method utilizes infrared spectroscopy. It has been shown that for TCNQ there is an excellent linear correlation of the degree of ionicity with the nitrile stretching frequency (ν_CN_), and that the frequencies are relatively insensitive to the crystal environment (Chappell *et al.*, 1981 ▸). In *D*_3*h*_-BTT·TCNQ, a frequency of 2218 cm^−1^ was observed, a slight decrease of ν_CN_ from that in neutral TCNQ. This frequency is identical to that of the *C*_3*h*_-BTT·TCNQ·toluene complex and correlates to a charge transfer ρ of 0.20 *e*. Based on both methods, the degree of charge transfer in the two TCNQ complexes is nearly identical. This is not surprising, inasmuch as the HOMO–LUMO gaps for *D*_3*h*_-BTT and *C*_3*h*_-BTT only differ by 0.2–0.3 eV (Guo *et al.*, 2011 ▸).

***D*** **_3_** ***_h_*** **-BTT·C_60_·Toluene**

**Figure 3:**
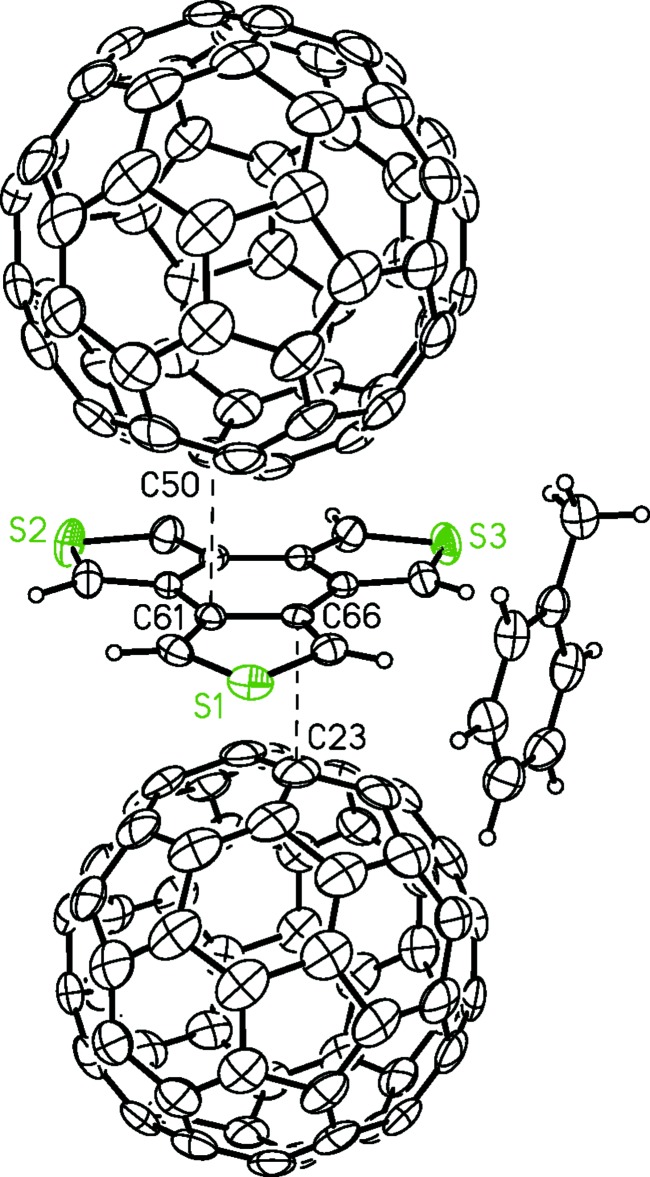
BTT-*D_3h_* ‘squeezed’ between two C_60_ mol­ecules in *D*_3h_-BTT·C_60_·toluene. The minor disordered fullerene moiety is omitted for clarity.

*D*_3*h*_-BTT and C_60_ form a 1:1 complex with the inclusion of a toluene mol­ecule, which was used as the solvent for CT formation. The shortest donor–acceptor contact is between one of the carbons of the central ‘benzene’ ring of *D*_3*h*_-BTT and C_60_ at 3.014 (6) Å (C50⋯C61). This distance is 0.39 Å shorter than the sum of the van der Waals radii for the two carbon atoms. On the other side of the BTT mol­ecule, a second C_60_ makes a contact of only 3.051 (6) Å to C66, the carbon adjacent to C61 (C23⋯C66) (Fig. 3 ▸). The C_60_ unit is clearly disordered. Initial refinement with *P*2_1_ as the space group led to elongated ellipsoids for the carbon atoms of C_60_. Refinement was noticeably improved and the ellipsoids became more reasonable in appearance using a model in which the C_60_ unit was disordered about a pseudo mirror plane.

We attempted to estimate the charge transfer by comparing the C—C and C=C bond-length variations between those of the of *D*_3*h*_-BTT donor itself and those in the CT complex. However, the bond-ratio results were not informative. We compared the IR spectra of the CT complex with those of the donor and acceptor. The IR spectrum of the CT complex is not a simple sum of the spectra of the donor and acceptor, suggesting that there is charge transfer, but no qu­anti­tative estimate can be drawn from the data. In addition, a prelim­inary measure of the magnetic susceptibility showed diamagnetism, which suggests a very low degree of charge transfer to be present.

## Supra­molecular features   

**Figure 4:**
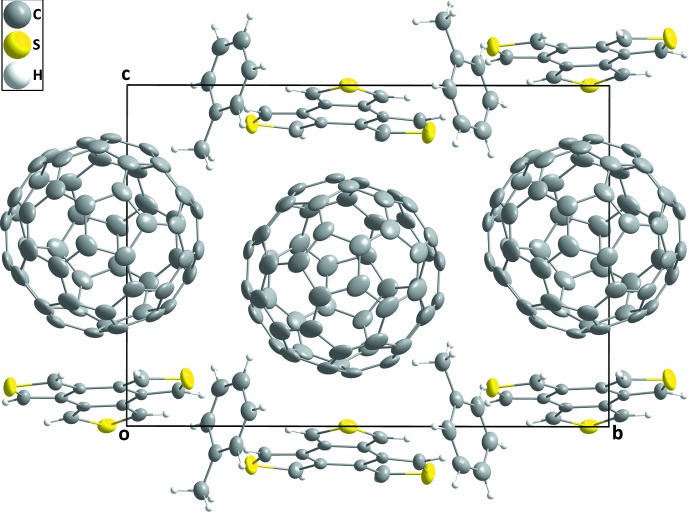
Packing of BTT-*D_3h_*·C_60_·toluene viewed along the *a*-axis direction. Minor disordered atoms are omitted for clarity.

In *D*_3*h*_-BTT·C_60_·toluene, the C_60_ mol­ecules form straight columns along the *a*-axis direction. These columns are sandwiched by corrugated sheets of *D*_3*h*_-BTT. Adjacent C_60_ columns form a zigzag pattern along the *b*-axis direction. The toluene mol­ecules reside as an array down the *a* axis in a pocket formed between the donor and acceptor. The toluene mol­ecule sits in an edge-to-face relationship with the π-system of the donor but it showed no particular close contact with either the donor or the acceptor (Figs. 4 ▸ and 5 ▸).

## Database survey   

An extensive search of the Cambridge Structural Database (Version 5.40, update of May 2019; Groom *et al.*, 2016 ▸) for close C_60_–arene contacts found only one example in which two C_60_ mol­ecules make contacts shorter than 3.05 Å to carbon atoms on both sides of an organic π-system. In that case (CSD refcode VOPNEV; Sun *et al.* 2014 ▸), two C_60_ mol­ecules touch a substituted ethyl­ene that lies on a center of inversion. The two (symmetry-related) contact distances are 3.013 Å. The present case, with contacts of 3.014 (6) and 3.051 (6) Å on the two sides of the central aromatic ring of the donor, yields the next smallest sum of contact distances 6.065 (12) Å] after the pair in VOPNEV (6.026 Å).

## Synthesis and crystallization   

*D*_3*h*_-BTT was prepared by a literature procedure (Hart *et al.*, 1978 ▸).

***D*** **_3_** ***_h_-*** **BTT·TCNQ**: A solution of *D*_3*h*_-BTT (10 mg, 4.1 mmol) in aceto­nitrile (3 mL) and a solution of TCNQ (8.3 mg, 4.1 mmol) in aceto­nitrile (3 mL) were mixed. This solution was then left to evaporate slowly in the dark at room temperature. After 30% of solvent had evaporated, dark needles formed in the test tube. We note that the color of TCNQ solution is green, but it turns dark immediately upon mixing with a solution of *D*_3*h*_-BTT, which is almost colorless.

***D*** **_3_** ***_h_-*** **BTT·C_60_·Toluene**: *D*_3*h*_-BTT (5 mg, 2.0 mmol) was dissolved in toluene (1.5 mL). C_60_ (14 mg, 4.0 mmol) was dissolved in toluene (4 mL) to give a dark-purple solution. C_60_ is sparingly soluble in toluene, and this solution was warmed and filtered before mixing with the solution of *D*_3*h*_-BTT. The mixture was warmed briefly, and then it was left to evaporate in the dark at room temperature. Dark, square plates of the CT complex formed upon complete solvent evaporation.

## Refinement   

Crystal data, data collection and structure refinement details are summarized in Table 1 ▸. In the final stages of the refinement of *D*_3_ *_h_-*BTT·C_60_·toluene, it became evident that there was disorder in the C_60_ portion. The refinement was continued using two orientations of the C_60_ portion, starting with idealized units and continuing with allowing them to ‘relax’ somewhat subject to the restraints ISOR 0.01 and SIMU 0.01 for all their carbon atoms. In addition, the geometries of the two orientations were restrained to be similar using a SAME instruction for the second component. The final refined occupancies for the two components are 0.766 (3) and 0.234 (3). Additionally, the structure was refined as two-component inversion twin. Hydrogen atoms in both structures were included as riding contributions with isotropic displacement parameters tied to those of the attached atoms with C—H distances of 0.93, 0.95 or 0.98 Å, and *U*_iso_(H) equal to 1.2 or 1.5 times *U*_eq_(C) of the carrier atom. Selected H atoms in *D*_3_ *_h_-*BTT·C_60_·toluene were freely refined (H67–H72).

## Supplementary Material

Crystal structure: contains datablock(s) I, II, global. DOI: 10.1107/S2056989019013161/zl2760sup1.cif

Structure factors: contains datablock(s) I. DOI: 10.1107/S2056989019013161/zl2760Isup2.hkl

Structure factors: contains datablock(s) II. DOI: 10.1107/S2056989019013161/zl2760IIsup3.hkl

CCDC references: 1955656, 1955655

Additional supporting information:  crystallographic information; 3D view; checkCIF report

## Figures and Tables

**Figure 5:**
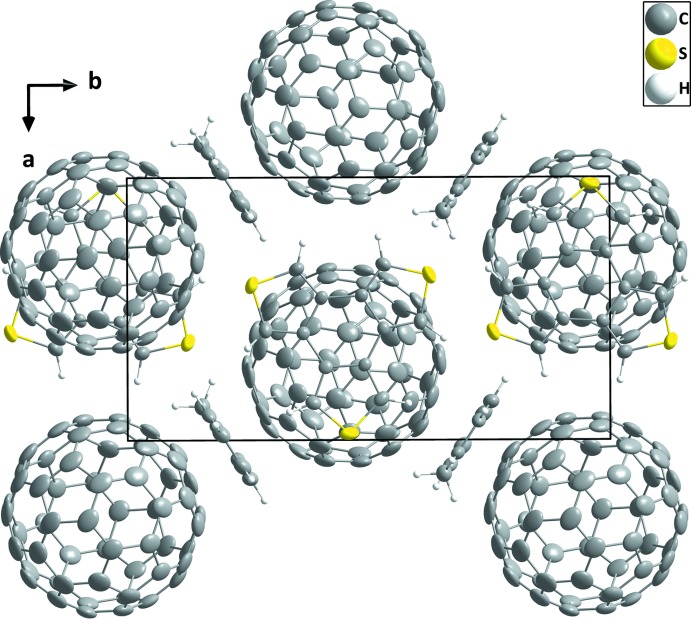
Packing of BTT-*D_3h_*·C_60_·toluene viewed along the *c*-axis direction. Minor disordered atoms are omitted for clarity.

**Table 1:** Experimental details

	*D*_3*h*_-BTT·TCNQ	(*D*_3*h*_-BTT·C_60_·toluene)
Crystal data		
Chemical formula	C_12_H_6_S_3_·C_12_H_4_N_4_	C_12_H_6_S_3_·C_60_·C_7_H_8_
*M*_r_	450.54	1059.08
Crystal system, space group	Monoclinic, *P*2_1_/*n*	Monoclinic, *P*2_1_
Temperature (K)	298	150
*a*, *b*, *c* (Å)	14.2567 (3), 39.0280 (7), 15.2295 (3)	10.0139 (6), 17.4327 (10), 13.0286 (8)
β (°)	100.136 (1)	108.816 (2)
*V* (Å^3^)	8341.6 (3)	2152.8 (2)
*Z*	16	2
Radiation type	Cu *K*α	Cu *K*α
μ (mm^−1^)	3.41	2.05
Crystal size (mm)	0.37 × 0.11 × 0.07	0.33 × 0.30 × 0.03

Data collection		
Diffractometer	Bruker D8 VENTURE PHOTON 100 CMOS	Bruker D8 VENTURE PHOTON 100 CMOS
Absorption correction	Multi-scan (*SADABS*; Krause *et al.*, 2015 ▸)	Multi-scan (*SADABS*; Krause *et al.*, 2015 ▸)
*T*_min_, *T*_max_	0.37, 0.80	0.76, 0.94
No. of measured, independent and observed [*I* > 2σ(*I*)] reflections	69033, 15720, 11912	16787, 7520, 7066
*R*_int_	0.050	0.035
(sin θ/λ)_max_ (Å^−1^)	0.610	0.625

Refinement		
*R*[*F* ^2^ > 2σ(*F* ^2^)], *wR*(*F* ^2^), *S*	0.044, 0.112, 1.03	0.042, 0.112, 1.04
No. of reflections	15720	7520
No. of parameters	1118	1307
No. of restraints	0	3769
H-atom treatment	H-atom parameters constrained	H atoms treated by a mixture of independent and constrained refinement
Δρ_max_, Δρ_min_ (e Å^−3^)	0.47, −0.57	0.33, −0.26
Absolute structure	–	Refined as an inversion twin
Absolute structure parameter	–	0.36 (2)

Computer programs: *APEX3* and *SAINT* (Bruker, 2016 ▸), *SHELXT* (Sheldrick, 2015*a* ▸), *SHELXL2018/1* (Sheldrick, 2015*b* ▸), *DIAMOND* (Brandenburg & Putz, 2012 ▸) and *SHELXTL* (Sheldrick, 2008 ▸).

## supplementary crystallographic information

### Benzo[1,2-c:3,4-c':5,6-c'']trithiophene–7,7,8,8-tetracyanoquinodimethane (I) Crystal data

**Table:**

C_12_H_6_S_3_·C_12_H_4_N_4_	*F*(000) = 3680
*M**_r_* = 450.54	*D*_x_ = 1.435 Mg m^−^^3^
Monoclinic, *P*2_1_/*n*	Cu *K*α radiation, λ = 1.54178 Å
*a* = 14.2567 (3) Å	Cell parameters from 9404 reflections
*b* = 39.0280 (7) Å	θ = 3.3–69.9°
*c* = 15.2295 (3) Å	µ = 3.41 mm^−^^1^
β = 100.136 (1)°	*T* = 298 K
*V* = 8341.6 (3) Å^3^	Column, black
*Z* = 16	0.37 × 0.11 × 0.07 mm

### Benzo[1,2-c:3,4-c':5,6-c'']trithiophene–7,7,8,8-tetracyanoquinodimethane (I) Data collection

**Table:**

Bruker D8 VENTURE PHOTON 100 CMOS diffractometer	15720 independent reflections
Radiation source: INCOATEC IµS micro-focus source	11912 reflections with *I* > 2σ(*I*)
Mirror monochromator	*R*_int_ = 0.050
ω scans	θ_max_ = 70.1°, θ_min_ = 2.3°
Absorption correction: multi-scan (*SADABS*; Krause *et al.*, 2015)	*h* = −17→17
*T*_min_ = 0.37, *T*_max_ = 0.80	*k* = −47→47
69033 measured reflections	*l* = −18→18

### Benzo[1,2-c:3,4-c':5,6-c'']trithiophene–7,7,8,8-tetracyanoquinodimethane (I) Refinement

**Table:**

Refinement on *F*^2^	Secondary atom site location: difference Fourier map
Least-squares matrix: full	Hydrogen site location: inferred from neighbouring sites
*R*[*F*^2^ > 2σ(*F*^2^)] = 0.044	H-atom parameters constrained
*wR*(*F*^2^) = 0.112	*w* = 1/[σ^2^(*F*_o_^2^) + (0.0446*P*)^2^ + 3.140*P*] where *P* = (*F*_o_^2^ + 2*F*_c_^2^)/3
*S* = 1.03	(Δ/σ)_max_ = 0.001
15720 reflections	Δρ_max_ = 0.47 e Å^−^^3^
1118 parameters	Δρ_min_ = −0.57 e Å^−^^3^
0 restraints	Extinction correction: *SHELXL2018/1* (Sheldrick 2015b), Fc^*^=kFc[1+0.001xFc^2^λ^3^/sin(2θ)]^-1/4^
Primary atom site location: structure-invariant direct methods	Extinction coefficient: 0.00038 (2)

### Benzo[1,2-c:3,4-c':5,6-c'']trithiophene–7,7,8,8-tetracyanoquinodimethane (I) Special details

**Table:**

Geometry. All esds (except the esd in the dihedral angle between two l.s. planes) are estimated using the full covariance matrix. The cell esds are taken into account individually in the estimation of esds in distances, angles and torsion angles; correlations between esds in cell parameters are only used when they are defined by crystal symmetry. An approximate (isotropic) treatment of cell esds is used for estimating esds involving l.s. planes.
Refinement. Refinement of F^2^ against ALL reflections. The weighted R-factor wR and goodness of fit S are based on F^2^, conventional R-factors R are based on F, with F set to zero for negative F^2^. The threshold expression of F^2^ > 2sigma(F^2^) is used only for calculating R-factors(gt) etc. and is not relevant to the choice of reflections for refinement. R-factors based on F^2^ are statistically about twice as large as those based on F, and R- factors based on ALL data will be even larger.

### Benzo[1,2-c:3,4-c':5,6-c'']trithiophene–7,7,8,8-tetracyanoquinodimethane (I) Fractional atomic coordinates and isotropic or equivalent isotropic displacement parameters (Å^2^)

**Table:**

	*x*	*y*	*z*	*U*_iso_*/*U*_eq_	
S1	0.84055 (6)	0.39144 (2)	0.49559 (4)	0.0749 (2)	
S2	0.83062 (6)	0.50480 (2)	0.79935 (5)	0.06945 (19)	
S3	0.97852 (6)	0.35327 (2)	0.90678 (4)	0.06507 (18)	
C49	0.88457 (15)	0.39131 (6)	0.66490 (14)	0.0441 (5)	
C50	0.84862 (16)	0.42488 (6)	0.64055 (14)	0.0474 (5)	
C51	0.84686 (16)	0.45071 (6)	0.70970 (15)	0.0469 (5)	
C52	0.87809 (16)	0.44200 (5)	0.80186 (15)	0.0449 (5)	
C53	0.91165 (15)	0.40751 (5)	0.82677 (14)	0.0426 (5)	
C54	0.91554 (15)	0.38240 (5)	0.75838 (14)	0.0421 (5)	
C55	0.88401 (19)	0.37071 (7)	0.59225 (16)	0.0574 (6)	
H55	0.904884	0.348090	0.595366	0.069*	
C56	0.8224 (2)	0.42822 (7)	0.55060 (17)	0.0648 (7)	
H56	0.797477	0.448215	0.522476	0.078*	
C57	0.81906 (19)	0.48428 (6)	0.70003 (18)	0.0602 (6)	
H57	0.796495	0.494606	0.645348	0.072*	
C58	0.87337 (19)	0.46929 (6)	0.85742 (17)	0.0567 (6)	
H58	0.891301	0.468482	0.919130	0.068*	
C59	0.94359 (18)	0.39493 (6)	0.91007 (15)	0.0546 (6)	
H59	0.946034	0.407519	0.962270	0.066*	
C60	0.95148 (18)	0.35195 (6)	0.79388 (16)	0.0533 (6)	
H60	0.960098	0.332747	0.760026	0.064*	
N1	0.2591 (2)	0.43047 (6)	0.33043 (17)	0.0854 (8)	
N2	0.1476 (2)	0.42514 (7)	0.04713 (18)	0.0909 (9)	
N3	−0.00125 (19)	0.22220 (7)	0.09289 (17)	0.0787 (7)	
N4	0.08798 (18)	0.22423 (6)	0.37656 (17)	0.0734 (6)	
C1	0.2214 (2)	0.41435 (6)	0.27299 (17)	0.0598 (6)	
C2	0.17541 (18)	0.39441 (6)	0.19893 (16)	0.0524 (5)	
C3	0.1598 (2)	0.41159 (6)	0.11411 (18)	0.0626 (7)	
C4	0.14938 (16)	0.36084 (6)	0.20744 (15)	0.0468 (5)	
C5	0.16313 (16)	0.34399 (6)	0.29316 (14)	0.0478 (5)	
H5	0.190536	0.356008	0.343960	0.057*	
C6	0.13704 (16)	0.31135 (6)	0.30055 (15)	0.0477 (5)	
H6	0.145591	0.301148	0.356583	0.057*	
C7	0.09582 (15)	0.29167 (6)	0.22303 (14)	0.0443 (5)	
C8	0.08292 (16)	0.30846 (6)	0.13756 (15)	0.0500 (5)	
H8	0.056495	0.296381	0.086545	0.060*	
C9	0.10842 (16)	0.34123 (6)	0.13049 (15)	0.0504 (5)	
H9	0.099390	0.351491	0.074529	0.060*	
C10	0.06977 (16)	0.25799 (6)	0.22977 (16)	0.0489 (5)	
C11	0.08081 (18)	0.23987 (6)	0.31212 (18)	0.0543 (6)	
C12	0.02986 (18)	0.23842 (6)	0.15278 (17)	0.0553 (6)	
S4	0.89924 (5)	0.26364 (2)	0.44991 (4)	0.06419 (18)	
S5	0.89951 (5)	0.38717 (2)	0.17110 (5)	0.06666 (19)	
S6	0.74345 (6)	0.23932 (2)	0.03393 (4)	0.06735 (19)	
C61	0.81337 (15)	0.26352 (5)	0.18689 (14)	0.0422 (5)	
C62	0.84797 (15)	0.26921 (5)	0.28141 (14)	0.0426 (5)	
C63	0.88671 (15)	0.30189 (5)	0.31239 (14)	0.0442 (5)	
C64	0.88625 (15)	0.33001 (5)	0.24852 (15)	0.0444 (5)	
C65	0.85021 (15)	0.32449 (5)	0.15600 (15)	0.0448 (5)	
C66	0.81512 (15)	0.29083 (5)	0.12405 (14)	0.0436 (5)	
C67	0.85041 (17)	0.24630 (6)	0.35012 (15)	0.0521 (5)	
H67	0.827963	0.223919	0.342858	0.063*	
C68	0.91787 (18)	0.30205 (6)	0.40273 (16)	0.0561 (6)	
H68	0.945840	0.320873	0.434512	0.067*	
C69	0.91584 (18)	0.36313 (6)	0.26580 (19)	0.0588 (6)	
H69	0.941595	0.371414	0.322144	0.071*	
C70	0.85299 (17)	0.35368 (6)	0.10659 (18)	0.0552 (6)	
H70	0.832013	0.354996	0.045306	0.066*	
C71	0.77918 (19)	0.28094 (6)	0.03891 (16)	0.0578 (6)	
H71	0.774746	0.295247	−0.010492	0.069*	
C72	0.77642 (18)	0.23411 (6)	0.14584 (16)	0.0527 (6)	
H72	0.770085	0.213677	0.175673	0.063*	
N5	0.0065 (3)	0.55087 (8)	0.6289 (2)	0.1136 (11)	
N6	0.1009 (3)	0.53006 (8)	0.9116 (2)	0.1167 (12)	
N7	0.20976 (18)	0.32793 (5)	0.79665 (16)	0.0666 (6)	
N8	0.1443 (3)	0.35063 (8)	0.51307 (18)	0.1000 (10)	
C13	0.0348 (2)	0.53153 (7)	0.6840 (2)	0.0784 (9)	
C14	0.0698 (2)	0.50701 (6)	0.7522 (2)	0.0634 (7)	
C15	0.0866 (3)	0.51971 (7)	0.8411 (2)	0.0794 (9)	
C16	0.08646 (17)	0.47346 (6)	0.73223 (17)	0.0534 (6)	
C17	0.12012 (17)	0.44925 (6)	0.80242 (16)	0.0516 (5)	
H17	0.128674	0.456315	0.861610	0.062*	
C18	0.13914 (16)	0.41679 (6)	0.78343 (15)	0.0485 (5)	
H18	0.159753	0.401558	0.829768	0.058*	
C19	0.12832 (16)	0.40499 (6)	0.69263 (15)	0.0470 (5)	
C20	0.09238 (17)	0.42897 (6)	0.62247 (16)	0.0546 (6)	
H20	0.083080	0.421884	0.563266	0.065*	
C21	0.07233 (18)	0.46135 (6)	0.64201 (17)	0.0585 (6)	
H21	0.048665	0.476314	0.595894	0.070*	
C22	0.15167 (17)	0.37213 (6)	0.67347 (16)	0.0505 (5)	
C23	0.18467 (17)	0.34764 (6)	0.74240 (17)	0.0516 (5)	
C24	0.1464 (2)	0.36010 (7)	0.58407 (19)	0.0651 (7)	
S7	0.45070 (5)	0.33951 (2)	0.74534 (5)	0.05738 (16)	
S8	0.28412 (6)	0.47068 (2)	0.51692 (4)	0.06695 (19)	
S9	0.37613 (6)	0.47960 (2)	0.94335 (5)	0.0723 (2)	
C73	0.40777 (15)	0.40025 (5)	0.78651 (14)	0.0438 (5)	
C74	0.38254 (15)	0.39779 (5)	0.69105 (14)	0.0420 (5)	
C75	0.34447 (16)	0.42764 (5)	0.63916 (14)	0.0434 (5)	
C76	0.33288 (16)	0.45949 (5)	0.68329 (15)	0.0447 (5)	
C77	0.35436 (16)	0.46162 (5)	0.78007 (15)	0.0447 (5)	
C78	0.39107 (16)	0.43212 (6)	0.83154 (14)	0.0453 (5)	
C79	0.44628 (17)	0.37044 (6)	0.82335 (16)	0.0526 (6)	
H79	0.467668	0.367361	0.884131	0.063*	
C80	0.40149 (17)	0.36586 (6)	0.66086 (16)	0.0508 (5)	
H80	0.388896	0.359386	0.601168	0.061*	
C81	0.31984 (19)	0.43039 (6)	0.54856 (16)	0.0552 (6)	
H81	0.322358	0.412370	0.509078	0.066*	
C82	0.30046 (19)	0.48487 (6)	0.62408 (16)	0.0563 (6)	
H82	0.288735	0.507220	0.640364	0.068*	
C83	0.34359 (19)	0.48894 (6)	0.83340 (17)	0.0581 (6)	
H83	0.320994	0.510243	0.811916	0.070*	
C84	0.40570 (19)	0.43872 (7)	0.92096 (16)	0.0581 (6)	
H84	0.429132	0.422706	0.964562	0.070*	
N9	0.72274 (19)	0.34886 (6)	0.87636 (18)	0.0768 (7)	
N10	0.6576 (2)	0.34271 (6)	0.58647 (18)	0.0805 (7)	
N11	0.4812 (2)	0.54939 (6)	0.57408 (15)	0.0775 (7)	
N12	0.5506 (2)	0.55348 (6)	0.85586 (16)	0.0849 (8)	
C25	0.69586 (18)	0.36239 (6)	0.81055 (18)	0.0550 (6)	
C26	0.66168 (16)	0.37944 (6)	0.72796 (16)	0.0494 (5)	
C27	0.65884 (18)	0.35911 (6)	0.64893 (18)	0.0566 (6)	
C28	0.63244 (15)	0.41299 (5)	0.72477 (14)	0.0436 (5)	
C29	0.59686 (16)	0.42969 (5)	0.64131 (15)	0.0474 (5)	
H29	0.593363	0.417564	0.588260	0.057*	
C30	0.56859 (16)	0.46263 (5)	0.63902 (14)	0.0465 (5)	
H30	0.545631	0.472905	0.584367	0.056*	
C31	0.57332 (16)	0.48223 (5)	0.71983 (14)	0.0437 (5)	
C32	0.60779 (17)	0.46534 (6)	0.80345 (14)	0.0487 (5)	
H32	0.610491	0.477350	0.856574	0.058*	
C33	0.63603 (17)	0.43252 (6)	0.80562 (15)	0.0489 (5)	
H33	0.658353	0.422179	0.860313	0.059*	
C34	0.54562 (17)	0.51595 (5)	0.71695 (14)	0.0466 (5)	
C35	0.51007 (19)	0.53373 (6)	0.63650 (15)	0.0526 (6)	
C36	0.5492 (2)	0.53617 (6)	0.79555 (16)	0.0573 (6)	
S10	0.46319 (6)	0.35807 (2)	0.41424 (5)	0.0803 (2)	
S11	0.28792 (5)	0.21874 (2)	0.22857 (6)	0.0743 (2)	
S12	0.31096 (6)	0.35350 (2)	−0.00664 (4)	0.06436 (18)	
C85	0.36342 (15)	0.33969 (5)	0.15849 (14)	0.0439 (5)	
C86	0.39724 (16)	0.34094 (6)	0.25398 (15)	0.0476 (5)	
C87	0.39319 (17)	0.31054 (6)	0.30705 (16)	0.0525 (6)	
C88	0.35319 (16)	0.27905 (6)	0.26459 (17)	0.0516 (6)	
C89	0.31797 (16)	0.27810 (5)	0.17079 (16)	0.0469 (5)	
C90	0.32373 (15)	0.30874 (5)	0.11668 (15)	0.0442 (5)	
C91	0.43345 (19)	0.36839 (7)	0.30488 (17)	0.0602 (6)	
H91	0.440845	0.390139	0.282081	0.072*	
C92	0.4277 (2)	0.31677 (8)	0.39511 (18)	0.0712 (8)	
H92	0.431389	0.300254	0.439596	0.085*	
C93	0.34102 (19)	0.24779 (7)	0.3036 (2)	0.0685 (8)	
H93	0.359843	0.243241	0.364044	0.082*	
C94	0.28058 (18)	0.24686 (6)	0.14272 (19)	0.0593 (6)	
H94	0.254412	0.241696	0.083892	0.071*	
C95	0.29310 (18)	0.31296 (6)	0.02765 (16)	0.0533 (6)	
H95	0.265496	0.295631	−0.010200	0.064*	
C96	0.36077 (18)	0.36586 (6)	0.09813 (16)	0.0550 (6)	
H96	0.383578	0.387824	0.112810	0.066*	
N13	0.7165 (2)	0.43205 (6)	0.3054 (2)	0.0913 (8)	
N14	0.6326 (2)	0.41094 (8)	0.0233 (2)	0.0984 (9)	
N15	0.5910 (2)	0.23135 (7)	0.42366 (18)	0.0826 (7)	
N16	0.51627 (17)	0.20817 (6)	0.14068 (16)	0.0693 (6)	
C37	0.6901 (2)	0.41224 (6)	0.25165 (19)	0.0623 (7)	
C38	0.65620 (17)	0.38776 (6)	0.18357 (17)	0.0529 (6)	
C39	0.6429 (2)	0.40055 (7)	0.0943 (2)	0.0650 (7)	
C40	0.63911 (16)	0.35419 (6)	0.20266 (16)	0.0474 (5)	
C41	0.65520 (16)	0.34202 (6)	0.29330 (15)	0.0496 (5)	
H41	0.679160	0.356958	0.339393	0.060*	
C42	0.63616 (16)	0.30945 (6)	0.31230 (16)	0.0502 (5)	
H42	0.647118	0.302169	0.371363	0.060*	
C43	0.59909 (15)	0.28564 (6)	0.24280 (15)	0.0466 (5)	
C44	0.58559 (16)	0.29757 (6)	0.15162 (16)	0.0511 (5)	
H44	0.563387	0.282474	0.105374	0.061*	
C45	0.60463 (17)	0.33007 (6)	0.13286 (16)	0.0512 (5)	
H45	0.595423	0.337202	0.073698	0.061*	
C46	0.57684 (16)	0.25262 (6)	0.26187 (16)	0.0497 (5)	
C47	0.58572 (19)	0.24062 (6)	0.35168 (19)	0.0580 (6)	
C48	0.54294 (18)	0.22815 (6)	0.19383 (18)	0.0533 (6)	

### Benzo[1,2-c:3,4-c':5,6-c'']trithiophene–7,7,8,8-tetracyanoquinodimethane (I) Atomic displacement parameters (Å^2^)

**Table:**

	*U*^11^	*U*^22^	*U*^33^	*U*^12^	*U*^13^	*U*^23^
S1	0.0959 (6)	0.0910 (5)	0.0356 (3)	0.0076 (4)	0.0058 (3)	−0.0051 (3)
S2	0.0870 (5)	0.0435 (3)	0.0730 (4)	0.0036 (3)	0.0007 (4)	−0.0051 (3)
S3	0.0829 (5)	0.0601 (4)	0.0482 (3)	0.0125 (3)	0.0005 (3)	0.0126 (3)
C49	0.0428 (12)	0.0518 (12)	0.0378 (11)	−0.0036 (9)	0.0071 (9)	−0.0004 (9)
C50	0.0487 (13)	0.0562 (13)	0.0361 (11)	−0.0012 (10)	0.0046 (10)	0.0052 (9)
C51	0.0475 (12)	0.0469 (11)	0.0443 (12)	−0.0015 (9)	0.0029 (10)	0.0047 (9)
C52	0.0477 (12)	0.0442 (11)	0.0411 (11)	−0.0041 (9)	0.0027 (10)	0.0014 (9)
C53	0.0427 (11)	0.0460 (11)	0.0378 (11)	−0.0043 (9)	0.0033 (9)	0.0015 (9)
C54	0.0409 (11)	0.0444 (11)	0.0408 (11)	−0.0032 (9)	0.0068 (9)	0.0005 (9)
C55	0.0661 (16)	0.0644 (14)	0.0413 (12)	0.0023 (12)	0.0081 (12)	−0.0056 (11)
C56	0.0796 (19)	0.0694 (16)	0.0436 (13)	0.0074 (14)	0.0058 (13)	0.0087 (11)
C57	0.0686 (17)	0.0509 (13)	0.0564 (15)	0.0024 (12)	−0.0021 (13)	0.0075 (11)
C58	0.0677 (16)	0.0498 (12)	0.0493 (13)	−0.0018 (11)	0.0009 (12)	−0.0049 (10)
C59	0.0657 (16)	0.0573 (13)	0.0384 (12)	0.0002 (11)	0.0023 (11)	0.0017 (10)
C60	0.0602 (15)	0.0492 (12)	0.0502 (13)	0.0040 (11)	0.0090 (12)	0.0013 (10)
N1	0.118 (2)	0.0571 (13)	0.0684 (16)	−0.0002 (14)	−0.0178 (16)	−0.0066 (12)
N2	0.128 (3)	0.0706 (15)	0.0647 (16)	−0.0065 (15)	−0.0089 (16)	0.0093 (13)
N3	0.0828 (17)	0.0795 (16)	0.0707 (16)	−0.0114 (13)	0.0049 (14)	−0.0265 (13)
N4	0.0771 (17)	0.0714 (14)	0.0670 (16)	−0.0029 (12)	−0.0003 (13)	0.0032 (12)
C1	0.0738 (17)	0.0462 (12)	0.0530 (14)	0.0064 (12)	−0.0060 (13)	−0.0028 (11)
C2	0.0555 (14)	0.0518 (12)	0.0466 (13)	0.0067 (11)	0.0001 (11)	−0.0035 (10)
C3	0.0782 (19)	0.0515 (13)	0.0531 (15)	0.0019 (12)	−0.0023 (14)	−0.0025 (12)
C4	0.0419 (12)	0.0543 (12)	0.0424 (12)	0.0061 (10)	0.0022 (10)	−0.0067 (9)
C5	0.0501 (13)	0.0537 (12)	0.0362 (11)	0.0022 (10)	−0.0020 (10)	−0.0077 (9)
C6	0.0474 (13)	0.0572 (13)	0.0365 (11)	0.0030 (10)	0.0017 (10)	−0.0038 (9)
C7	0.0360 (11)	0.0532 (12)	0.0428 (12)	0.0032 (9)	0.0045 (9)	−0.0090 (9)
C8	0.0477 (13)	0.0620 (14)	0.0383 (11)	0.0014 (10)	0.0023 (10)	−0.0124 (10)
C9	0.0518 (13)	0.0584 (13)	0.0387 (12)	0.0029 (11)	0.0016 (10)	−0.0047 (10)
C10	0.0415 (12)	0.0558 (13)	0.0485 (13)	0.0030 (10)	0.0049 (10)	−0.0075 (10)
C11	0.0500 (14)	0.0546 (13)	0.0558 (15)	−0.0024 (11)	0.0024 (12)	−0.0087 (12)
C12	0.0543 (14)	0.0556 (13)	0.0550 (14)	−0.0030 (11)	0.0068 (12)	−0.0129 (11)
S4	0.0804 (5)	0.0743 (4)	0.0371 (3)	−0.0056 (3)	0.0084 (3)	0.0057 (3)
S5	0.0687 (4)	0.0444 (3)	0.0905 (5)	0.0013 (3)	0.0239 (4)	0.0107 (3)
S6	0.0868 (5)	0.0655 (4)	0.0451 (3)	−0.0074 (3)	−0.0013 (3)	−0.0053 (3)
C61	0.0402 (11)	0.0472 (11)	0.0393 (11)	0.0045 (9)	0.0075 (9)	0.0038 (9)
C62	0.0410 (11)	0.0481 (11)	0.0394 (11)	0.0025 (9)	0.0087 (9)	0.0047 (9)
C63	0.0433 (12)	0.0486 (11)	0.0421 (11)	0.0031 (9)	0.0110 (10)	−0.0018 (9)
C64	0.0406 (11)	0.0427 (11)	0.0519 (13)	0.0023 (9)	0.0135 (10)	0.0007 (9)
C65	0.0406 (11)	0.0465 (11)	0.0492 (12)	0.0079 (9)	0.0138 (10)	0.0066 (9)
C66	0.0439 (12)	0.0488 (11)	0.0388 (11)	0.0057 (9)	0.0090 (10)	0.0038 (9)
C67	0.0579 (14)	0.0560 (13)	0.0424 (12)	−0.0052 (11)	0.0084 (11)	0.0036 (10)
C68	0.0620 (15)	0.0609 (14)	0.0454 (13)	−0.0052 (12)	0.0097 (12)	−0.0073 (11)
C69	0.0576 (15)	0.0495 (13)	0.0710 (17)	0.0007 (11)	0.0158 (13)	−0.0050 (11)
C70	0.0545 (14)	0.0533 (13)	0.0601 (15)	0.0077 (11)	0.0162 (12)	0.0132 (11)
C71	0.0711 (17)	0.0596 (14)	0.0413 (12)	0.0049 (12)	0.0063 (12)	0.0076 (10)
C72	0.0603 (15)	0.0505 (12)	0.0465 (13)	−0.0023 (11)	0.0068 (11)	0.0024 (10)
N5	0.133 (3)	0.0793 (19)	0.126 (3)	0.0074 (18)	0.016 (2)	0.0433 (19)
N6	0.189 (4)	0.0684 (17)	0.090 (2)	0.001 (2)	0.015 (2)	−0.0122 (16)
N7	0.0797 (16)	0.0533 (12)	0.0656 (14)	0.0075 (11)	0.0090 (12)	−0.0024 (11)
N8	0.145 (3)	0.099 (2)	0.0585 (16)	−0.0124 (19)	0.0256 (17)	−0.0192 (14)
C13	0.089 (2)	0.0539 (15)	0.093 (2)	−0.0007 (14)	0.0186 (18)	0.0172 (15)
C14	0.0673 (17)	0.0497 (13)	0.0735 (18)	−0.0021 (12)	0.0133 (14)	0.0069 (12)
C15	0.109 (3)	0.0457 (14)	0.083 (2)	0.0016 (15)	0.015 (2)	0.0017 (14)
C16	0.0507 (13)	0.0489 (12)	0.0610 (15)	−0.0041 (10)	0.0106 (12)	0.0049 (11)
C17	0.0582 (14)	0.0479 (12)	0.0478 (13)	−0.0053 (10)	0.0069 (11)	−0.0007 (10)
C18	0.0515 (13)	0.0479 (12)	0.0453 (12)	−0.0044 (10)	0.0060 (11)	0.0038 (9)
C19	0.0442 (12)	0.0492 (12)	0.0475 (12)	−0.0081 (9)	0.0082 (10)	0.0009 (9)
C20	0.0546 (14)	0.0617 (14)	0.0461 (13)	−0.0045 (11)	0.0052 (11)	0.0031 (11)
C21	0.0609 (15)	0.0561 (14)	0.0563 (15)	−0.0041 (11)	0.0047 (12)	0.0134 (11)
C22	0.0503 (13)	0.0542 (13)	0.0470 (13)	−0.0066 (10)	0.0082 (11)	−0.0032 (10)
C23	0.0533 (14)	0.0485 (12)	0.0529 (14)	−0.0037 (10)	0.0092 (12)	−0.0095 (11)
C24	0.0800 (19)	0.0609 (15)	0.0547 (16)	−0.0079 (13)	0.0127 (14)	−0.0061 (12)
S7	0.0606 (4)	0.0472 (3)	0.0654 (4)	0.0073 (3)	0.0142 (3)	0.0076 (3)
S8	0.0949 (5)	0.0567 (3)	0.0443 (3)	0.0029 (3)	−0.0015 (3)	0.0117 (3)
S9	0.0988 (6)	0.0692 (4)	0.0477 (4)	−0.0023 (4)	0.0098 (4)	−0.0153 (3)
C73	0.0418 (11)	0.0475 (11)	0.0423 (11)	−0.0017 (9)	0.0081 (10)	0.0051 (9)
C74	0.0413 (11)	0.0447 (11)	0.0402 (11)	−0.0021 (9)	0.0074 (9)	0.0016 (9)
C75	0.0445 (12)	0.0459 (11)	0.0390 (11)	−0.0040 (9)	0.0049 (10)	0.0023 (9)
C76	0.0469 (12)	0.0439 (11)	0.0421 (11)	−0.0056 (9)	0.0044 (10)	0.0008 (9)
C77	0.0448 (12)	0.0461 (11)	0.0427 (12)	−0.0064 (9)	0.0060 (10)	−0.0016 (9)
C78	0.0462 (12)	0.0509 (12)	0.0387 (11)	−0.0044 (9)	0.0075 (10)	0.0000 (9)
C79	0.0541 (14)	0.0563 (13)	0.0469 (13)	0.0031 (11)	0.0081 (11)	0.0105 (10)
C80	0.0557 (14)	0.0492 (12)	0.0477 (13)	0.0007 (10)	0.0098 (11)	−0.0008 (10)
C81	0.0694 (16)	0.0526 (13)	0.0412 (12)	−0.0012 (11)	0.0035 (12)	0.0000 (10)
C82	0.0706 (16)	0.0445 (12)	0.0513 (14)	0.0000 (11)	0.0035 (12)	0.0043 (10)
C83	0.0708 (17)	0.0488 (13)	0.0540 (14)	−0.0026 (11)	0.0093 (13)	−0.0058 (11)
C84	0.0674 (16)	0.0660 (15)	0.0394 (12)	−0.0004 (12)	0.0051 (12)	0.0010 (11)
N9	0.0838 (17)	0.0633 (13)	0.0770 (17)	0.0007 (12)	−0.0036 (14)	0.0205 (12)
N10	0.0923 (19)	0.0686 (15)	0.0800 (17)	0.0004 (13)	0.0132 (15)	−0.0177 (13)
N11	0.114 (2)	0.0651 (14)	0.0492 (13)	0.0132 (13)	0.0023 (13)	0.0043 (11)
N12	0.147 (3)	0.0539 (13)	0.0531 (14)	−0.0025 (14)	0.0143 (15)	−0.0099 (11)
C25	0.0553 (14)	0.0446 (12)	0.0631 (16)	−0.0009 (10)	0.0049 (12)	0.0059 (11)
C26	0.0471 (13)	0.0460 (12)	0.0546 (14)	−0.0015 (10)	0.0072 (11)	0.0037 (10)
C27	0.0569 (15)	0.0479 (12)	0.0631 (16)	0.0002 (11)	0.0052 (13)	−0.0013 (12)
C28	0.0432 (12)	0.0439 (11)	0.0433 (12)	−0.0020 (9)	0.0066 (10)	0.0019 (9)
C29	0.0539 (13)	0.0472 (12)	0.0404 (11)	−0.0007 (10)	0.0062 (10)	−0.0039 (9)
C30	0.0560 (13)	0.0461 (11)	0.0354 (11)	0.0004 (10)	0.0027 (10)	0.0001 (9)
C31	0.0486 (12)	0.0431 (11)	0.0394 (11)	−0.0031 (9)	0.0074 (10)	−0.0008 (9)
C32	0.0609 (14)	0.0501 (12)	0.0344 (11)	−0.0042 (10)	0.0062 (10)	−0.0015 (9)
C33	0.0569 (14)	0.0519 (12)	0.0363 (11)	−0.0028 (10)	0.0042 (10)	0.0057 (9)
C34	0.0571 (14)	0.0445 (11)	0.0370 (11)	−0.0037 (10)	0.0048 (10)	−0.0005 (9)
C35	0.0709 (16)	0.0454 (12)	0.0405 (12)	0.0008 (11)	0.0069 (12)	−0.0038 (10)
C36	0.0838 (18)	0.0417 (11)	0.0450 (13)	−0.0029 (11)	0.0075 (13)	0.0001 (10)
S10	0.0899 (5)	0.1019 (6)	0.0447 (4)	0.0193 (4)	−0.0004 (4)	−0.0171 (4)
S11	0.0696 (4)	0.0508 (3)	0.1102 (6)	0.0055 (3)	0.0370 (4)	0.0210 (4)
S12	0.0869 (5)	0.0653 (4)	0.0415 (3)	−0.0021 (3)	0.0129 (3)	0.0090 (3)
C85	0.0443 (12)	0.0466 (11)	0.0421 (12)	0.0053 (9)	0.0117 (10)	0.0011 (9)
C86	0.0475 (13)	0.0540 (12)	0.0415 (12)	0.0084 (10)	0.0083 (10)	−0.0006 (10)
C87	0.0473 (13)	0.0680 (15)	0.0431 (12)	0.0147 (11)	0.0104 (11)	0.0086 (11)
C88	0.0440 (12)	0.0562 (13)	0.0579 (14)	0.0115 (10)	0.0179 (11)	0.0166 (11)
C89	0.0434 (12)	0.0468 (11)	0.0540 (13)	0.0073 (9)	0.0178 (11)	0.0047 (10)
C90	0.0433 (12)	0.0466 (11)	0.0451 (12)	0.0036 (9)	0.0144 (10)	0.0010 (9)
C91	0.0644 (16)	0.0628 (15)	0.0512 (14)	0.0118 (12)	0.0042 (13)	−0.0071 (11)
C92	0.0765 (19)	0.092 (2)	0.0452 (14)	0.0206 (16)	0.0110 (14)	0.0117 (13)
C93	0.0572 (16)	0.0784 (18)	0.0741 (18)	0.0187 (13)	0.0230 (14)	0.0324 (15)
C94	0.0568 (15)	0.0504 (13)	0.0756 (18)	0.0011 (11)	0.0247 (14)	0.0027 (12)
C95	0.0615 (15)	0.0548 (13)	0.0447 (12)	−0.0025 (11)	0.0118 (11)	−0.0039 (10)
C96	0.0662 (16)	0.0499 (12)	0.0498 (13)	−0.0014 (11)	0.0129 (12)	0.0044 (10)
N13	0.128 (2)	0.0527 (13)	0.090 (2)	0.0060 (14)	0.0113 (18)	−0.0093 (13)
N14	0.117 (2)	0.106 (2)	0.0738 (18)	0.0246 (18)	0.0201 (17)	0.0331 (16)
N15	0.103 (2)	0.0782 (16)	0.0676 (16)	0.0159 (14)	0.0169 (15)	0.0172 (13)
N16	0.0773 (16)	0.0559 (12)	0.0716 (15)	−0.0093 (11)	0.0048 (13)	0.0017 (11)
C37	0.0772 (18)	0.0440 (13)	0.0659 (17)	0.0103 (12)	0.0129 (14)	0.0057 (12)
C38	0.0542 (14)	0.0507 (12)	0.0544 (14)	0.0094 (10)	0.0106 (12)	0.0050 (10)
C39	0.0689 (17)	0.0607 (15)	0.0662 (17)	0.0121 (13)	0.0142 (14)	0.0124 (13)
C40	0.0421 (12)	0.0489 (12)	0.0515 (13)	0.0074 (9)	0.0090 (10)	0.0016 (10)
C41	0.0503 (13)	0.0503 (12)	0.0462 (12)	0.0052 (10)	0.0030 (11)	−0.0012 (10)
C42	0.0482 (13)	0.0531 (13)	0.0471 (13)	0.0059 (10)	0.0023 (11)	0.0055 (10)
C43	0.0395 (11)	0.0485 (12)	0.0516 (13)	0.0066 (9)	0.0078 (10)	0.0018 (10)
C44	0.0488 (13)	0.0538 (13)	0.0495 (13)	0.0038 (10)	0.0052 (11)	−0.0030 (10)
C45	0.0519 (14)	0.0559 (13)	0.0449 (12)	0.0062 (10)	0.0059 (11)	0.0038 (10)
C46	0.0441 (12)	0.0494 (12)	0.0557 (14)	0.0064 (10)	0.0088 (11)	0.0044 (10)
C47	0.0600 (15)	0.0503 (13)	0.0629 (16)	0.0077 (11)	0.0089 (13)	0.0074 (12)
C48	0.0516 (14)	0.0481 (12)	0.0601 (15)	0.0001 (10)	0.0089 (12)	0.0094 (11)

### Benzo[1,2-c:3,4-c':5,6-c'']trithiophene–7,7,8,8-tetracyanoquinodimethane (I) Geometric parameters (Å, º)

**Table:**

S1—C55	1.699 (3)	S7—C80	1.699 (2)
S1—C56	1.705 (3)	S7—C79	1.703 (3)
S2—C57	1.694 (3)	S8—C81	1.696 (2)
S2—C58	1.699 (3)	S8—C82	1.700 (3)
S3—C60	1.695 (2)	S9—C83	1.697 (3)
S3—C59	1.704 (2)	S9—C84	1.700 (3)
C49—C55	1.367 (3)	C73—C79	1.365 (3)
C49—C50	1.432 (3)	C73—C74	1.438 (3)
C49—C54	1.457 (3)	C73—C78	1.460 (3)
C50—C56	1.361 (3)	C74—C80	1.372 (3)
C50—C51	1.461 (3)	C74—C75	1.458 (3)
C51—C57	1.369 (3)	C75—C81	1.367 (3)
C51—C52	1.437 (3)	C75—C76	1.436 (3)
C52—C58	1.369 (3)	C76—C82	1.364 (3)
C52—C53	1.456 (3)	C76—C77	1.454 (3)
C53—C59	1.362 (3)	C77—C83	1.365 (3)
C53—C54	1.438 (3)	C77—C78	1.439 (3)
C54—C60	1.367 (3)	C78—C84	1.365 (3)
C55—H55	0.9300	C79—H79	0.9300
C56—H56	0.9300	C80—H80	0.9300
C57—H57	0.9300	C81—H81	0.9300
C58—H58	0.9300	C82—H82	0.9300
C59—H59	0.9300	C83—H83	0.9300
C60—H60	0.9300	C84—H84	0.9300
N1—C1	1.133 (3)	N9—C25	1.138 (3)
N2—C3	1.135 (3)	N10—C27	1.144 (3)
N3—C12	1.134 (3)	N11—C35	1.144 (3)
N4—C11	1.145 (3)	N12—C36	1.138 (3)
C1—C2	1.431 (3)	C25—C26	1.431 (3)
C2—C4	1.374 (3)	C26—C28	1.372 (3)
C2—C3	1.437 (4)	C26—C27	1.436 (3)
C4—C9	1.435 (3)	C28—C29	1.439 (3)
C4—C5	1.444 (3)	C28—C33	1.441 (3)
C5—C6	1.337 (3)	C29—C30	1.346 (3)
C5—H5	0.9300	C29—H29	0.9300
C6—C7	1.444 (3)	C30—C31	1.441 (3)
C6—H6	0.9300	C30—H30	0.9300
C7—C10	1.375 (3)	C31—C34	1.373 (3)
C7—C8	1.440 (3)	C31—C32	1.441 (3)
C8—C9	1.339 (3)	C32—C33	1.341 (3)
C8—H8	0.9300	C32—H32	0.9300
C9—H9	0.9300	C33—H33	0.9300
C10—C11	1.424 (4)	C34—C35	1.422 (3)
C10—C12	1.431 (3)	C34—C36	1.427 (3)
S4—C67	1.697 (2)	S10—C91	1.693 (3)
S4—C68	1.703 (3)	S10—C92	1.699 (3)
S5—C70	1.698 (3)	S11—C93	1.691 (3)
S5—C69	1.702 (3)	S11—C94	1.696 (3)
S6—C72	1.699 (2)	S12—C96	1.698 (3)
S6—C71	1.700 (3)	S12—C95	1.699 (2)
C61—C72	1.368 (3)	C85—C96	1.370 (3)
C61—C66	1.436 (3)	C85—C90	1.435 (3)
C61—C62	1.454 (3)	C85—C86	1.450 (3)
C62—C67	1.372 (3)	C86—C91	1.369 (3)
C62—C63	1.436 (3)	C86—C87	1.442 (3)
C63—C68	1.369 (3)	C87—C92	1.367 (4)
C63—C64	1.466 (3)	C87—C88	1.457 (4)
C64—C69	1.371 (3)	C88—C93	1.381 (3)
C64—C65	1.429 (3)	C88—C89	1.429 (3)
C65—C70	1.370 (3)	C89—C94	1.369 (3)
C65—C66	1.458 (3)	C89—C90	1.463 (3)
C66—C71	1.363 (3)	C90—C95	1.359 (3)
C67—H67	0.9300	C91—H91	0.9300
C68—H68	0.9300	C92—H92	0.9300
C69—H69	0.9300	C93—H93	0.9300
C70—H70	0.9300	C94—H94	0.9300
C71—H71	0.9300	C95—H95	0.9300
C72—H72	0.9300	C96—H96	0.9300
N5—C13	1.148 (4)	N13—C37	1.141 (4)
N6—C15	1.132 (4)	N14—C39	1.139 (4)
N7—C23	1.139 (3)	N15—C47	1.144 (3)
N8—C24	1.138 (4)	N16—C48	1.140 (3)
C13—C14	1.436 (4)	C37—C38	1.430 (4)
C14—C16	1.374 (3)	C38—C40	1.373 (3)
C14—C15	1.422 (4)	C38—C39	1.430 (4)
C16—C21	1.433 (4)	C40—C41	1.439 (3)
C16—C17	1.444 (3)	C40—C45	1.440 (3)
C17—C18	1.338 (3)	C41—C42	1.342 (3)
C17—H17	0.9300	C41—H41	0.9300
C18—C19	1.440 (3)	C42—C43	1.437 (3)
C18—H18	0.9300	C42—H42	0.9300
C19—C22	1.369 (3)	C43—C46	1.370 (3)
C19—C20	1.445 (3)	C43—C44	1.445 (3)
C20—C21	1.340 (3)	C44—C45	1.338 (3)
C20—H20	0.9300	C44—H44	0.9300
C21—H21	0.9300	C45—H45	0.9300
C22—C24	1.429 (3)	C46—C48	1.429 (4)
C22—C23	1.436 (3)	C46—C47	1.430 (3)
			
C55—S1—C56	92.23 (12)	C80—S7—C79	92.19 (11)
C57—S2—C58	92.80 (12)	C81—S8—C82	92.62 (12)
C60—S3—C59	92.48 (11)	C83—S9—C84	92.19 (12)
C55—C49—C50	112.2 (2)	C79—C73—C74	111.7 (2)
C55—C49—C54	127.5 (2)	C79—C73—C78	128.3 (2)
C50—C49—C54	120.31 (19)	C74—C73—C78	119.96 (19)
C56—C50—C49	111.8 (2)	C80—C74—C73	111.75 (19)
C56—C50—C51	128.5 (2)	C80—C74—C75	128.4 (2)
C49—C50—C51	119.69 (19)	C73—C74—C75	119.85 (19)
C57—C51—C52	111.4 (2)	C81—C75—C76	112.0 (2)
C57—C51—C50	128.6 (2)	C81—C75—C74	128.0 (2)
C52—C51—C50	119.96 (19)	C76—C75—C74	120.02 (19)
C58—C52—C51	112.3 (2)	C82—C76—C75	111.9 (2)
C58—C52—C53	127.4 (2)	C82—C76—C77	127.9 (2)
C51—C52—C53	120.28 (19)	C75—C76—C77	120.19 (19)
C59—C53—C54	112.3 (2)	C83—C77—C78	111.7 (2)
C59—C53—C52	128.1 (2)	C83—C77—C76	128.5 (2)
C54—C53—C52	119.60 (19)	C78—C77—C76	119.86 (19)
C60—C54—C53	111.4 (2)	C84—C78—C77	111.7 (2)
C60—C54—C49	128.5 (2)	C84—C78—C73	128.3 (2)
C53—C54—C49	120.10 (19)	C77—C78—C73	119.98 (19)
C49—C55—S1	111.75 (19)	C73—C79—S7	112.29 (18)
C49—C55—H55	124.1	C73—C79—H79	123.9
S1—C55—H55	124.1	S7—C79—H79	123.9
C50—C56—S1	112.0 (2)	C74—C80—S7	112.05 (18)
C50—C56—H56	124.0	C74—C80—H80	124.0
S1—C56—H56	124.0	S7—C80—H80	124.0
C51—C57—S2	112.05 (19)	C75—C81—S8	111.75 (18)
C51—C57—H57	124.0	C75—C81—H81	124.1
S2—C57—H57	124.0	S8—C81—H81	124.1
C52—C58—S2	111.41 (19)	C76—C82—S8	111.78 (18)
C52—C58—H58	124.3	C76—C82—H82	124.1
S2—C58—H58	124.3	S8—C82—H82	124.1
C53—C59—S3	111.61 (18)	C77—C83—S9	112.30 (19)
C53—C59—H59	124.2	C77—C83—H83	123.9
S3—C59—H59	124.2	S9—C83—H83	123.9
C54—C60—S3	112.15 (18)	C78—C84—S9	112.16 (19)
C54—C60—H60	123.9	C78—C84—H84	123.9
S3—C60—H60	123.9	S9—C84—H84	123.9
N1—C1—C2	178.4 (3)	N9—C25—C26	179.7 (3)
C4—C2—C1	122.5 (2)	C28—C26—C25	122.0 (2)
C4—C2—C3	121.9 (2)	C28—C26—C27	122.3 (2)
C1—C2—C3	115.6 (2)	C25—C26—C27	115.8 (2)
N2—C3—C2	179.9 (4)	N10—C27—C26	179.1 (3)
C2—C4—C9	120.6 (2)	C26—C28—C29	121.4 (2)
C2—C4—C5	121.6 (2)	C26—C28—C33	120.6 (2)
C9—C4—C5	117.8 (2)	C29—C28—C33	117.96 (19)
C6—C5—C4	121.0 (2)	C30—C29—C28	120.9 (2)
C6—C5—H5	119.5	C30—C29—H29	119.6
C4—C5—H5	119.5	C28—C29—H29	119.6
C5—C6—C7	121.1 (2)	C29—C30—C31	121.1 (2)
C5—C6—H6	119.4	C29—C30—H30	119.5
C7—C6—H6	119.4	C31—C30—H30	119.5
C10—C7—C8	120.6 (2)	C34—C31—C30	120.8 (2)
C10—C7—C6	121.6 (2)	C34—C31—C32	121.2 (2)
C8—C7—C6	117.8 (2)	C30—C31—C32	118.00 (19)
C9—C8—C7	120.9 (2)	C33—C32—C31	120.8 (2)
C9—C8—H8	119.5	C33—C32—H32	119.6
C7—C8—H8	119.5	C31—C32—H32	119.6
C8—C9—C4	121.4 (2)	C32—C33—C28	121.2 (2)
C8—C9—H9	119.3	C32—C33—H33	119.4
C4—C9—H9	119.3	C28—C33—H33	119.4
C7—C10—C11	123.4 (2)	C31—C34—C35	123.5 (2)
C7—C10—C12	121.5 (2)	C31—C34—C36	122.4 (2)
C11—C10—C12	115.0 (2)	C35—C34—C36	114.1 (2)
N4—C11—C10	177.3 (3)	N11—C35—C34	176.8 (3)
N3—C12—C10	178.3 (3)	N12—C36—C34	177.0 (3)
C67—S4—C68	92.47 (12)	C91—S10—C92	92.38 (13)
C70—S5—C69	92.71 (12)	C93—S11—C94	92.66 (13)
C72—S6—C71	92.39 (12)	C96—S12—C95	92.07 (12)
C72—C61—C66	111.6 (2)	C96—C85—C90	111.6 (2)
C72—C61—C62	128.1 (2)	C96—C85—C86	127.8 (2)
C66—C61—C62	120.26 (19)	C90—C85—C86	120.58 (19)
C67—C62—C63	111.8 (2)	C91—C86—C87	112.0 (2)
C67—C62—C61	127.9 (2)	C91—C86—C85	128.3 (2)
C63—C62—C61	120.31 (19)	C87—C86—C85	119.7 (2)
C68—C63—C62	111.9 (2)	C92—C87—C86	111.1 (2)
C68—C63—C64	128.8 (2)	C92—C87—C88	129.1 (2)
C62—C63—C64	119.4 (2)	C86—C87—C88	119.7 (2)
C69—C64—C65	112.1 (2)	C93—C88—C89	111.0 (2)
C69—C64—C63	127.8 (2)	C93—C88—C87	128.6 (3)
C65—C64—C63	120.03 (19)	C89—C88—C87	120.5 (2)
C70—C65—C64	112.1 (2)	C94—C89—C88	112.6 (2)
C70—C65—C66	127.4 (2)	C94—C89—C90	127.6 (2)
C64—C65—C66	120.51 (19)	C88—C89—C90	119.8 (2)
C71—C66—C61	112.1 (2)	C95—C90—C85	111.9 (2)
C71—C66—C65	128.4 (2)	C95—C90—C89	128.4 (2)
C61—C66—C65	119.4 (2)	C85—C90—C89	119.7 (2)
C62—C67—S4	111.98 (18)	C86—C91—S10	112.0 (2)
C62—C67—H67	124.0	C86—C91—H91	124.0
S4—C67—H67	124.0	S10—C91—H91	124.0
C63—C68—S4	111.88 (18)	C87—C92—S10	112.5 (2)
C63—C68—H68	124.1	C87—C92—H92	123.8
S4—C68—H68	124.1	S10—C92—H92	123.8
C64—C69—S5	111.4 (2)	C88—C93—S11	112.2 (2)
C64—C69—H69	124.3	C88—C93—H93	123.9
S5—C69—H69	124.3	S11—C93—H93	123.9
C65—C70—S5	111.6 (2)	C89—C94—S11	111.6 (2)
C65—C70—H70	124.2	C89—C94—H94	124.2
S5—C70—H70	124.2	S11—C94—H94	124.2
C66—C71—S6	111.86 (18)	C90—C95—S12	112.35 (18)
C66—C71—H71	124.1	C90—C95—H95	123.8
S6—C71—H71	124.1	S12—C95—H95	123.8
C61—C72—S6	112.00 (17)	C85—C96—S12	112.11 (18)
C61—C72—H72	124.0	C85—C96—H96	123.9
S6—C72—H72	124.0	S12—C96—H96	123.9
N5—C13—C14	179.3 (4)	N13—C37—C38	179.3 (3)
C16—C14—C15	122.4 (3)	C40—C38—C39	122.4 (2)
C16—C14—C13	121.8 (3)	C40—C38—C37	122.3 (2)
C15—C14—C13	115.8 (3)	C39—C38—C37	115.3 (2)
N6—C15—C14	179.1 (4)	N14—C39—C38	179.5 (4)
C14—C16—C21	121.7 (2)	C38—C40—C41	120.9 (2)
C14—C16—C17	120.5 (2)	C38—C40—C45	121.2 (2)
C21—C16—C17	117.8 (2)	C41—C40—C45	117.9 (2)
C18—C17—C16	120.9 (2)	C42—C41—C40	121.0 (2)
C18—C17—H17	119.6	C42—C41—H41	119.5
C16—C17—H17	119.6	C40—C41—H41	119.5
C17—C18—C19	121.3 (2)	C41—C42—C43	121.1 (2)
C17—C18—H18	119.4	C41—C42—H42	119.5
C19—C18—H18	119.4	C43—C42—H42	119.5
C22—C19—C18	121.0 (2)	C46—C43—C42	121.4 (2)
C22—C19—C20	121.1 (2)	C46—C43—C44	120.7 (2)
C18—C19—C20	117.9 (2)	C42—C43—C44	117.9 (2)
C21—C20—C19	120.5 (2)	C45—C44—C43	120.9 (2)
C21—C20—H20	119.7	C45—C44—H44	119.6
C19—C20—H20	119.7	C43—C44—H44	119.6
C20—C21—C16	121.6 (2)	C44—C45—C40	121.2 (2)
C20—C21—H21	119.2	C44—C45—H45	119.4
C16—C21—H21	119.2	C40—C45—H45	119.4
C19—C22—C24	122.4 (2)	C43—C46—C48	122.4 (2)
C19—C22—C23	121.9 (2)	C43—C46—C47	121.7 (2)
C24—C22—C23	115.7 (2)	C48—C46—C47	115.9 (2)
N7—C23—C22	179.0 (3)	N15—C47—C46	178.5 (3)
N8—C24—C22	178.5 (4)	N16—C48—C46	178.8 (3)
			
C55—C49—C50—C56	0.0 (3)	C79—C73—C74—C80	1.3 (3)
C54—C49—C50—C56	−179.3 (2)	C78—C73—C74—C80	−178.7 (2)
C55—C49—C50—C51	−178.1 (2)	C79—C73—C74—C75	−177.1 (2)
C54—C49—C50—C51	2.6 (3)	C78—C73—C74—C75	2.9 (3)
C56—C50—C51—C57	−0.5 (4)	C80—C74—C75—C81	−0.5 (4)
C49—C50—C51—C57	177.2 (2)	C73—C74—C75—C81	177.6 (2)
C56—C50—C51—C52	−179.8 (2)	C80—C74—C75—C76	−177.6 (2)
C49—C50—C51—C52	−2.1 (3)	C73—C74—C75—C76	0.5 (3)
C57—C51—C52—C58	−0.5 (3)	C81—C75—C76—C82	−0.3 (3)
C50—C51—C52—C58	178.9 (2)	C74—C75—C76—C82	177.3 (2)
C57—C51—C52—C53	−179.4 (2)	C81—C75—C76—C77	179.2 (2)
C50—C51—C52—C53	0.1 (3)	C74—C75—C76—C77	−3.2 (3)
C58—C52—C53—C59	1.3 (4)	C82—C76—C77—C83	2.7 (4)
C51—C52—C53—C59	180.0 (2)	C75—C76—C77—C83	−176.7 (2)
C58—C52—C53—C54	−177.1 (2)	C82—C76—C77—C78	−178.0 (2)
C51—C52—C53—C54	1.5 (3)	C75—C76—C77—C78	2.6 (3)
C59—C53—C54—C60	−0.6 (3)	C83—C77—C78—C84	0.2 (3)
C52—C53—C54—C60	178.1 (2)	C76—C77—C78—C84	−179.1 (2)
C59—C53—C54—C49	−179.7 (2)	C83—C77—C78—C73	−179.9 (2)
C52—C53—C54—C49	−1.0 (3)	C76—C77—C78—C73	0.7 (3)
C55—C49—C54—C60	0.8 (4)	C79—C73—C78—C84	−3.7 (4)
C50—C49—C54—C60	−180.0 (2)	C74—C73—C78—C84	176.4 (2)
C55—C49—C54—C53	179.7 (2)	C79—C73—C78—C77	176.5 (2)
C50—C49—C54—C53	−1.1 (3)	C74—C73—C78—C77	−3.5 (3)
C50—C49—C55—S1	−0.1 (3)	C74—C73—C79—S7	−1.0 (3)
C54—C49—C55—S1	179.20 (18)	C78—C73—C79—S7	179.09 (18)
C56—S1—C55—C49	0.1 (2)	C80—S7—C79—C73	0.31 (19)
C49—C50—C56—S1	0.0 (3)	C73—C74—C80—S7	−1.1 (2)
C51—C50—C56—S1	177.9 (2)	C75—C74—C80—S7	177.14 (18)
C55—S1—C56—C50	−0.1 (2)	C79—S7—C80—C74	0.47 (19)
C52—C51—C57—S2	0.2 (3)	C76—C75—C81—S8	0.4 (3)
C50—C51—C57—S2	−179.2 (2)	C74—C75—C81—S8	−176.93 (19)
C58—S2—C57—C51	0.2 (2)	C82—S8—C81—C75	−0.4 (2)
C51—C52—C58—S2	0.7 (3)	C75—C76—C82—S8	0.0 (3)
C53—C52—C58—S2	179.40 (19)	C77—C76—C82—S8	−179.44 (19)
C57—S2—C58—C52	−0.5 (2)	C81—S8—C82—C76	0.2 (2)
C54—C53—C59—S3	0.2 (3)	C78—C77—C83—S9	−0.3 (3)
C52—C53—C59—S3	−178.40 (19)	C76—C77—C83—S9	178.99 (19)
C60—S3—C59—C53	0.2 (2)	C84—S9—C83—C77	0.2 (2)
C53—C54—C60—S3	0.8 (3)	C77—C78—C84—S9	0.0 (3)
C49—C54—C60—S3	179.78 (18)	C73—C78—C84—S9	−179.92 (19)
C59—S3—C60—C54	−0.6 (2)	C83—S9—C84—C78	−0.1 (2)
C1—C2—C4—C9	−176.4 (2)	C25—C26—C28—C29	−179.1 (2)
C3—C2—C4—C9	2.3 (4)	C27—C26—C28—C29	0.2 (3)
C1—C2—C4—C5	2.8 (4)	C25—C26—C28—C33	0.6 (3)
C3—C2—C4—C5	−178.5 (2)	C27—C26—C28—C33	179.8 (2)
C2—C4—C5—C6	179.7 (2)	C26—C28—C29—C30	−179.9 (2)
C9—C4—C5—C6	−1.1 (3)	C33—C28—C29—C30	0.5 (3)
C4—C5—C6—C7	1.1 (3)	C28—C29—C30—C31	0.2 (3)
C5—C6—C7—C10	179.2 (2)	C29—C30—C31—C34	179.1 (2)
C5—C6—C7—C8	−0.6 (3)	C29—C30—C31—C32	−1.0 (3)
C10—C7—C8—C9	−179.8 (2)	C34—C31—C32—C33	−179.0 (2)
C6—C7—C8—C9	0.0 (3)	C30—C31—C32—C33	1.1 (3)
C7—C8—C9—C4	0.0 (3)	C31—C32—C33—C28	−0.3 (4)
C2—C4—C9—C8	179.8 (2)	C26—C28—C33—C32	179.9 (2)
C5—C4—C9—C8	0.5 (3)	C29—C28—C33—C32	−0.5 (3)
C8—C7—C10—C11	179.9 (2)	C30—C31—C34—C35	0.7 (4)
C6—C7—C10—C11	0.0 (3)	C32—C31—C34—C35	−179.2 (2)
C8—C7—C10—C12	0.5 (3)	C30—C31—C34—C36	179.9 (2)
C6—C7—C10—C12	−179.3 (2)	C32—C31—C34—C36	0.0 (4)
C72—C61—C62—C67	−0.5 (4)	C96—C85—C86—C91	0.3 (4)
C66—C61—C62—C67	179.2 (2)	C90—C85—C86—C91	−177.3 (2)
C72—C61—C62—C63	178.5 (2)	C96—C85—C86—C87	179.2 (2)
C66—C61—C62—C63	−1.8 (3)	C90—C85—C86—C87	1.6 (3)
C67—C62—C63—C68	0.9 (3)	C91—C86—C87—C92	−0.7 (3)
C61—C62—C63—C68	−178.3 (2)	C85—C86—C87—C92	−179.8 (2)
C67—C62—C63—C64	−177.5 (2)	C91—C86—C87—C88	177.7 (2)
C61—C62—C63—C64	3.3 (3)	C85—C86—C87—C88	−1.4 (3)
C68—C63—C64—C69	−1.2 (4)	C92—C87—C88—C93	−0.3 (4)
C62—C63—C64—C69	176.9 (2)	C86—C87—C88—C93	−178.4 (2)
C68—C63—C64—C65	−180.0 (2)	C92—C87—C88—C89	178.0 (2)
C62—C63—C64—C65	−1.9 (3)	C86—C87—C88—C89	0.0 (3)
C69—C64—C65—C70	−0.5 (3)	C93—C88—C89—C94	0.4 (3)
C63—C64—C65—C70	178.43 (19)	C87—C88—C89—C94	−178.2 (2)
C69—C64—C65—C66	−180.0 (2)	C93—C88—C89—C90	179.8 (2)
C63—C64—C65—C66	−1.0 (3)	C87—C88—C89—C90	1.2 (3)
C72—C61—C66—C71	0.3 (3)	C96—C85—C90—C95	−0.1 (3)
C62—C61—C66—C71	−179.4 (2)	C86—C85—C90—C95	177.8 (2)
C72—C61—C66—C65	178.6 (2)	C96—C85—C90—C89	−178.4 (2)
C62—C61—C66—C65	−1.1 (3)	C86—C85—C90—C89	−0.4 (3)
C70—C65—C66—C71	1.1 (4)	C94—C89—C90—C95	0.4 (4)
C64—C65—C66—C71	−179.5 (2)	C88—C89—C90—C95	−178.9 (2)
C70—C65—C66—C61	−176.8 (2)	C94—C89—C90—C85	178.4 (2)
C64—C65—C66—C61	2.6 (3)	C88—C89—C90—C85	−0.9 (3)
C63—C62—C67—S4	−0.5 (3)	C87—C86—C91—S10	0.5 (3)
C61—C62—C67—S4	178.62 (18)	C85—C86—C91—S10	179.49 (19)
C68—S4—C67—C62	0.0 (2)	C92—S10—C91—C86	−0.2 (2)
C62—C63—C68—S4	−0.9 (3)	C86—C87—C92—S10	0.6 (3)
C64—C63—C68—S4	177.30 (18)	C88—C87—C92—S10	−177.6 (2)
C67—S4—C68—C63	0.5 (2)	C91—S10—C92—C87	−0.2 (2)
C65—C64—C69—S5	0.3 (2)	C89—C88—C93—S11	−0.5 (3)
C63—C64—C69—S5	−178.57 (18)	C87—C88—C93—S11	178.00 (19)
C70—S5—C69—C64	0.00 (19)	C94—S11—C93—C88	0.4 (2)
C64—C65—C70—S5	0.5 (2)	C88—C89—C94—S11	−0.1 (3)
C66—C65—C70—S5	179.94 (18)	C90—C89—C94—S11	−179.47 (18)
C69—S5—C70—C65	−0.31 (19)	C93—S11—C94—C89	−0.14 (19)
C61—C66—C71—S6	0.0 (3)	C85—C90—C95—S12	−0.1 (3)
C65—C66—C71—S6	−178.00 (18)	C89—C90—C95—S12	177.99 (18)
C72—S6—C71—C66	−0.3 (2)	C96—S12—C95—C90	0.3 (2)
C66—C61—C72—S6	−0.5 (3)	C90—C85—C96—S12	0.3 (3)
C62—C61—C72—S6	179.15 (18)	C86—C85—C96—S12	−177.48 (18)
C71—S6—C72—C61	0.5 (2)	C95—S12—C96—C85	−0.3 (2)
C15—C14—C16—C21	−177.8 (3)	C39—C38—C40—C41	178.7 (2)
C13—C14—C16—C21	1.8 (4)	C37—C38—C40—C41	−0.3 (4)
C15—C14—C16—C17	1.5 (4)	C39—C38—C40—C45	−1.3 (4)
C13—C14—C16—C17	−178.9 (3)	C37—C38—C40—C45	179.7 (2)
C14—C16—C17—C18	−178.0 (2)	C38—C40—C41—C42	178.2 (2)
C21—C16—C17—C18	1.3 (4)	C45—C40—C41—C42	−1.8 (3)
C16—C17—C18—C19	1.0 (4)	C40—C41—C42—C43	0.0 (3)
C17—C18—C19—C22	177.3 (2)	C41—C42—C43—C46	−178.1 (2)
C17—C18—C19—C20	−2.5 (3)	C41—C42—C43—C44	1.8 (3)
C22—C19—C20—C21	−178.1 (2)	C46—C43—C44—C45	178.0 (2)
C18—C19—C20—C21	1.7 (3)	C42—C43—C44—C45	−1.8 (3)
C19—C20—C21—C16	0.6 (4)	C43—C44—C45—C40	0.0 (3)
C14—C16—C21—C20	177.2 (2)	C38—C40—C45—C44	−178.2 (2)
C17—C16—C21—C20	−2.1 (4)	C41—C40—C45—C44	1.8 (3)
C18—C19—C22—C24	−176.8 (2)	C42—C43—C46—C48	−177.8 (2)
C20—C19—C22—C24	3.0 (4)	C44—C43—C46—C48	2.3 (3)
C18—C19—C22—C23	2.2 (3)	C42—C43—C46—C47	2.5 (3)
C20—C19—C22—C23	−178.0 (2)	C44—C43—C46—C47	−177.3 (2)

### Benzo[1,2-c:3,4-c':5,6-c'']trithiophene–7,7,8,8-tetracyanoquinodimethane (I) Hydrogen-bond geometry (Å, º)

**Table:**

*D*—H···*A*	*D*—H	H···*A*	*D*···*A*	*D*—H···*A*
C57—H57···S8^i^	0.93	2.87	3.803 (3)	175
C58—H58···N6^ii^	0.93	2.56	3.472 (4)	166
C82—H82···N13^i^	0.93	2.52	3.438 (3)	171
C92—H92···N3^iii^	0.93	2.52	3.367 (4)	151
C96—H96···N12^i^	0.93	2.49	3.419 (3)	177

Symmetry codes: (i) −*x*+1, −*y*+1, −*z*+1; (ii) −*x*+1, −*y*+1, −*z*+2; (iii) *x*+1/2, −*y*+1/2, *z*+1/2.

### Benzo[1,2-c:3,4-c':5,6-c'']trithiophene–\ buckminsterfullerene–toluene (II) Crystal data

**Table:**

C_12_H_6_S_3_·C_60_·C_7_H_8_	*F*(000) = 1072
*M**_r_* = 1059.08	*D*_x_ = 1.634 Mg m^−^^3^
Monoclinic, *P*2_1_	Cu *K*α radiation, λ = 1.54178 Å
*a* = 10.0139 (6) Å	Cell parameters from 9987 reflections
*b* = 17.4327 (10) Å	θ = 3.6–74.5°
*c* = 13.0286 (8) Å	µ = 2.05 mm^−^^1^
β = 108.816 (2)°	*T* = 150 K
*V* = 2152.8 (2) Å^3^	Plate, dark red
*Z* = 2	0.33 × 0.30 × 0.03 mm

### Benzo[1,2-c:3,4-c':5,6-c'']trithiophene–\ buckminsterfullerene–toluene (II) Data collection

**Table:**

Bruker D8 VENTURE PHOTON 100 CMOS diffractometer	7520 independent reflections
Radiation source: INCOATEC IµS micro–focus source	7066 reflections with *I* > 2σ(*I*)
Mirror monochromator	*R*_int_ = 0.035
Detector resolution: 10.4167 pixels mm^-1^	θ_max_ = 74.5°, θ_min_ = 3.6°
ω scans	*h* = −12→12
Absorption correction: multi-scan (*SADABS*; Krause *et al.*, 2015)	*k* = −20→21
*T*_min_ = 0.76, *T*_max_ = 0.94	*l* = −15→16
16787 measured reflections	

### Benzo[1,2-c:3,4-c':5,6-c'']trithiophene–\ buckminsterfullerene–toluene (II) Refinement

**Table:**

Refinement on *F*^2^	Hydrogen site location: mixed
Least-squares matrix: full	H atoms treated by a mixture of independent and constrained refinement
*R*[*F*^2^ > 2σ(*F*^2^)] = 0.042	*w* = 1/[σ^2^(*F*_o_^2^) + (0.064*P*)^2^ + 0.870*P*] where *P* = (*F*_o_^2^ + 2*F*_c_^2^)/3
*wR*(*F*^2^) = 0.112	(Δ/σ)_max_ = 0.001
*S* = 1.04	Δρ_max_ = 0.33 e Å^−^^3^
7520 reflections	Δρ_min_ = −0.26 e Å^−^^3^
1307 parameters	Extinction correction: *SHELXL2018/1* (Sheldrick, 2015*b*), Fc^*^=kFc[1+0.001xFc^2^λ^3^/sin(2θ)]^-1/4^
3769 restraints	Extinction coefficient: 0.0009 (2)
Primary atom site location: dual	Absolute structure: Refined as an inversion twin
Secondary atom site location: difference Fourier map	Absolute structure parameter: 0.36 (2)

### Benzo[1,2-c:3,4-c':5,6-c'']trithiophene–\ buckminsterfullerene–toluene (II) Special details

**Table:**

Geometry. All esds (except the esd in the dihedral angle between two l.s. planes) are estimated using the full covariance matrix. The cell esds are taken into account individually in the estimation of esds in distances, angles and torsion angles; correlations between esds in cell parameters are only used when they are defined by crystal symmetry. An approximate (isotropic) treatment of cell esds is used for estimating esds involving l.s. planes.
Refinement. Refinement of F^2^ against ALL reflections. The weighted R-factor wR and goodness of fit S are based on F^2^, conventional R-factors R are based on F, with F set to zero for negative F^2^. The threshold expression of F^2^ > 2sigma(F^2^) is used only for calculating R-factors(gt) etc. and is not relevant to the choice of reflections for refinement. R-factors based on F^2^ are statistically about twice as large as those based on F, and R- factors based on ALL data will be even larger. The H-atoms of the lattice toluene molecule were included as riding contributions in idealized positions. The C60 unit is disordered over two sites in a 0.766 (3)/0.234 (3) ratio and was refined as a 2-component inversion twin with restraints that the geometries of the two components be comparable.

### Benzo[1,2-c:3,4-c':5,6-c'']trithiophene–\ buckminsterfullerene–toluene (II) Fractional atomic coordinates and isotropic or equivalent isotropic displacement parameters (Å^2^)

**Table:**

	*x*	*y*	*z*	*U*_iso_*/*U*_eq_	Occ. (<1)
C1	0.5825 (9)	0.6221 (5)	0.5098 (6)	0.0540 (17)	0.766 (3)
C2	0.4772 (8)	0.6029 (4)	0.4111 (6)	0.0492 (15)	0.766 (3)
C3	0.3890 (6)	0.5410 (4)	0.4041 (6)	0.0497 (14)	0.766 (3)
C4	0.4054 (7)	0.4963 (5)	0.5022 (6)	0.0463 (15)	0.766 (3)
C5	0.5083 (8)	0.5143 (4)	0.5987 (6)	0.0467 (14)	0.766 (3)
C6	0.5993 (8)	0.5784 (4)	0.6035 (6)	0.0505 (15)	0.766 (3)
C7	0.5334 (13)	0.6162 (9)	0.3226 (10)	0.0520 (17)	0.766 (3)
C8	0.5013 (9)	0.5674 (6)	0.2342 (8)	0.0450 (16)	0.766 (3)
C9	0.4092 (10)	0.5025 (6)	0.2293 (8)	0.0478 (18)	0.766 (3)
C10	0.3548 (7)	0.4894 (5)	0.3134 (6)	0.0460 (15)	0.766 (3)
C11	0.3487 (6)	0.4131 (5)	0.3522 (6)	0.0486 (15)	0.766 (3)
C12	0.3816 (6)	0.4160 (5)	0.4710 (6)	0.0501 (15)	0.766 (3)
C13	0.4594 (8)	0.3582 (5)	0.5346 (7)	0.0533 (17)	0.766 (3)
C14	0.5665 (8)	0.3800 (5)	0.6359 (7)	0.0504 (17)	0.766 (3)
C15	0.6857 (8)	0.3265 (5)	0.6504 (6)	0.0489 (17)	0.766 (3)
C16	0.6506 (10)	0.2751 (6)	0.5593 (7)	0.0551 (18)	0.766 (3)
C17	0.5081 (8)	0.2966 (4)	0.4854 (7)	0.0569 (16)	0.766 (3)
C18	0.4778 (8)	0.2935 (4)	0.3755 (7)	0.0606 (18)	0.766 (3)
C19	0.3969 (8)	0.3520 (5)	0.3078 (7)	0.0561 (18)	0.766 (3)
C20	0.4537 (9)	0.3654 (5)	0.2185 (6)	0.0556 (18)	0.766 (3)
C21	0.4630 (14)	0.4399 (7)	0.1817 (13)	0.0547 (18)	0.766 (3)
C22	0.5858 (7)	0.4666 (5)	0.1567 (4)	0.0470 (14)	0.766 (3)
C23	0.6968 (8)	0.4171 (4)	0.1704 (5)	0.0535 (14)	0.766 (3)
C24	0.6885 (10)	0.3388 (5)	0.2091 (6)	0.0540 (17)	0.766 (3)
C25	0.5759 (9)	0.3132 (5)	0.2332 (6)	0.0572 (18)	0.766 (3)
C26	0.5918 (11)	0.2674 (6)	0.3299 (8)	0.062 (2)	0.766 (3)
C27	0.7265 (12)	0.2491 (8)	0.3999 (8)	0.063 (2)	0.766 (3)
C28	0.8479 (10)	0.2748 (5)	0.3730 (6)	0.0593 (18)	0.766 (3)
C29	0.8294 (9)	0.3174 (5)	0.2797 (7)	0.0598 (17)	0.766 (3)
C30	0.9217 (9)	0.3824 (5)	0.2855 (7)	0.0560 (16)	0.766 (3)
C31	0.8404 (9)	0.4437 (6)	0.2172 (8)	0.0526 (17)	0.766 (3)
C32	0.8646 (9)	0.5175 (6)	0.2486 (7)	0.0553 (18)	0.766 (3)
C33	0.9718 (8)	0.5404 (5)	0.3497 (7)	0.0563 (18)	0.766 (3)
C34	1.0504 (6)	0.4800 (6)	0.4123 (6)	0.0533 (15)	0.766 (3)
C35	1.0241 (8)	0.4006 (6)	0.3809 (7)	0.0526 (16)	0.766 (3)
C36	0.6798 (11)	0.6448 (8)	0.3674 (7)	0.0512 (18)	0.766 (3)
C37	0.9523 (9)	0.2934 (5)	0.4729 (7)	0.0568 (17)	0.766 (3)
C38	0.9006 (13)	0.2814 (9)	0.5634 (10)	0.0510 (17)	0.766 (3)
C39	0.7525 (12)	0.2525 (8)	0.5171 (8)	0.0564 (19)	0.766 (3)
C40	0.8212 (8)	0.3525 (5)	0.6940 (8)	0.0434 (17)	0.766 (3)
C41	0.9324 (9)	0.3312 (5)	0.6520 (8)	0.0445 (17)	0.766 (3)
C42	1.0434 (7)	0.3564 (5)	0.4805 (6)	0.0541 (15)	0.766 (3)
C43	1.0772 (7)	0.4077 (5)	0.5717 (6)	0.0488 (15)	0.766 (3)
C44	1.0824 (6)	0.4834 (5)	0.5312 (6)	0.0506 (15)	0.766 (3)
C45	1.0348 (7)	0.5456 (5)	0.5775 (6)	0.0543 (17)	0.766 (3)
C46	0.9526 (8)	0.6051 (4)	0.5090 (7)	0.0583 (17)	0.766 (3)
C47	0.9752 (8)	0.5329 (5)	0.6657 (6)	0.0519 (17)	0.766 (3)
C48	0.9687 (14)	0.4597 (6)	0.7037 (12)	0.0494 (17)	0.766 (3)
C49	0.8437 (7)	0.4315 (4)	0.7269 (4)	0.0432 (13)	0.766 (3)
C50	0.7336 (7)	0.4816 (4)	0.7135 (4)	0.0469 (13)	0.766 (3)
C51	0.5910 (9)	0.4562 (5)	0.6667 (7)	0.0474 (16)	0.766 (3)
C52	0.7417 (9)	0.5587 (4)	0.6758 (6)	0.0468 (15)	0.766 (3)
C53	0.8558 (8)	0.5851 (4)	0.6505 (6)	0.0514 (16)	0.766 (3)
C54	0.8382 (9)	0.6303 (5)	0.5554 (7)	0.0556 (18)	0.766 (3)
C55	0.7050 (11)	0.6483 (7)	0.4845 (8)	0.058 (2)	0.766 (3)
C56	0.7818 (10)	0.6222 (6)	0.3267 (7)	0.0552 (18)	0.766 (3)
C57	0.7463 (9)	0.5708 (5)	0.2337 (6)	0.0503 (17)	0.766 (3)
C58	0.9240 (8)	0.6016 (5)	0.3989 (7)	0.0583 (17)	0.766 (3)
C59	1.0225 (10)	0.3970 (5)	0.6554 (8)	0.0478 (18)	0.766 (3)
C60	0.6084 (8)	0.5454 (5)	0.1900 (7)	0.0462 (17)	0.766 (3)
C1B	0.7550 (19)	0.4162 (11)	0.7180 (14)	0.041 (3)	0.234 (3)
C2B	0.785 (3)	0.3427 (15)	0.686 (3)	0.042 (3)	0.234 (3)
C3B	0.901 (3)	0.3227 (19)	0.655 (3)	0.046 (4)	0.234 (3)
C4B	1.005 (4)	0.3828 (16)	0.664 (3)	0.048 (4)	0.234 (3)
C5B	0.978 (5)	0.4557 (16)	0.696 (4)	0.049 (4)	0.234 (3)
C6B	0.853 (2)	0.4763 (12)	0.7254 (15)	0.044 (3)	0.234 (3)
C7B	0.650 (3)	0.3128 (15)	0.622 (2)	0.053 (3)	0.234 (3)
C8B	0.638 (3)	0.270 (2)	0.530 (2)	0.058 (4)	0.234 (3)
C9B	0.759 (3)	0.251 (3)	0.499 (2)	0.057 (4)	0.234 (3)
C10B	0.889 (4)	0.278 (3)	0.560 (3)	0.051 (4)	0.234 (3)
C11B	0.990 (3)	0.3087 (14)	0.5117 (18)	0.055 (3)	0.234 (3)
C12B	1.057 (3)	0.3757 (13)	0.5728 (19)	0.052 (3)	0.234 (3)
C13B	1.089 (2)	0.4383 (14)	0.5221 (17)	0.056 (3)	0.234 (3)
C14B	1.067 (3)	0.5142 (14)	0.5608 (18)	0.052 (3)	0.234 (3)
C15B	1.017 (2)	0.5608 (12)	0.4604 (16)	0.058 (3)	0.234 (3)
C16B	1.004 (3)	0.5138 (14)	0.3669 (19)	0.056 (3)	0.234 (3)
C17B	1.049 (3)	0.4354 (14)	0.4067 (17)	0.050 (3)	0.234 (3)
C18B	0.986 (2)	0.3714 (13)	0.349 (2)	0.055 (3)	0.234 (3)
C19B	0.949 (2)	0.3064 (12)	0.3993 (17)	0.059 (3)	0.234 (3)
C20B	0.808 (2)	0.2760 (16)	0.338 (2)	0.060 (3)	0.234 (3)
C21B	0.706 (3)	0.249 (3)	0.381 (2)	0.060 (4)	0.234 (3)
C22B	0.560 (3)	0.270 (2)	0.342 (2)	0.059 (4)	0.234 (3)
C23B	0.525 (2)	0.3118 (14)	0.248 (2)	0.056 (3)	0.234 (3)
C24B	0.627 (2)	0.3417 (14)	0.198 (2)	0.053 (3)	0.234 (3)
C25B	0.763 (2)	0.3257 (13)	0.2389 (19)	0.055 (3)	0.234 (3)
C26B	0.864 (2)	0.3857 (13)	0.2431 (19)	0.056 (3)	0.234 (3)
C27B	0.826 (3)	0.4643 (14)	0.213 (3)	0.052 (3)	0.234 (3)
C28B	0.677 (2)	0.4785 (11)	0.1663 (15)	0.046 (3)	0.234 (3)
C29B	0.582 (2)	0.4188 (12)	0.1575 (15)	0.047 (3)	0.234 (3)
C30B	0.454 (5)	0.4355 (17)	0.185 (4)	0.051 (4)	0.234 (3)
C31B	0.422 (3)	0.3711 (15)	0.242 (2)	0.053 (3)	0.234 (3)
C32B	0.371 (3)	0.3803 (14)	0.3229 (18)	0.050 (3)	0.234 (3)
C33B	0.347 (2)	0.4552 (14)	0.3628 (16)	0.051 (3)	0.234 (3)
C34B	0.376 (3)	0.5172 (13)	0.3057 (19)	0.050 (3)	0.234 (3)
C35B	0.429 (4)	0.5078 (17)	0.216 (3)	0.048 (4)	0.234 (3)
C36B	0.535 (3)	0.3675 (15)	0.611 (2)	0.051 (3)	0.234 (3)
C37B	0.650 (3)	0.5535 (15)	0.196 (3)	0.043 (3)	0.234 (3)
C38B	0.778 (3)	0.5856 (15)	0.263 (2)	0.054 (3)	0.234 (3)
C39B	0.896 (3)	0.5305 (15)	0.275 (2)	0.052 (3)	0.234 (3)
C40B	0.917 (2)	0.6165 (13)	0.449 (2)	0.059 (3)	0.234 (3)
C41B	0.793 (3)	0.631 (2)	0.356 (2)	0.059 (4)	0.234 (3)
C42B	0.530 (3)	0.5710 (19)	0.225 (3)	0.048 (4)	0.234 (3)
C43B	0.543 (4)	0.617 (3)	0.320 (3)	0.051 (4)	0.234 (3)
C44B	0.444 (2)	0.5848 (13)	0.3676 (17)	0.054 (3)	0.234 (3)
C45B	0.482 (2)	0.5887 (12)	0.4826 (17)	0.056 (3)	0.234 (3)
C46B	0.450 (2)	0.5222 (13)	0.5355 (19)	0.050 (3)	0.234 (3)
C47B	0.625 (2)	0.6197 (15)	0.5438 (19)	0.055 (3)	0.234 (3)
C48B	0.727 (3)	0.647 (3)	0.503 (2)	0.054 (4)	0.234 (3)
C49B	0.875 (3)	0.6243 (19)	0.5425 (19)	0.055 (4)	0.234 (3)
C50B	0.911 (2)	0.5809 (13)	0.6355 (18)	0.053 (3)	0.234 (3)
C51B	1.014 (2)	0.5217 (14)	0.647 (2)	0.051 (3)	0.234 (3)
C52B	0.808 (2)	0.5537 (12)	0.6841 (18)	0.047 (3)	0.234 (3)
C53B	0.670 (2)	0.5713 (13)	0.6429 (18)	0.051 (3)	0.234 (3)
C54B	0.571 (2)	0.5106 (12)	0.6419 (18)	0.049 (3)	0.234 (3)
C55B	0.607 (3)	0.4339 (13)	0.673 (3)	0.044 (3)	0.234 (3)
C56B	0.429 (3)	0.3823 (14)	0.5176 (19)	0.053 (3)	0.234 (3)
C57B	0.416 (2)	0.3363 (11)	0.4237 (16)	0.058 (3)	0.234 (3)
C58B	0.385 (2)	0.4588 (14)	0.4783 (16)	0.046 (3)	0.234 (3)
C59B	0.669 (3)	0.647 (3)	0.384 (2)	0.052 (4)	0.234 (3)
C60B	0.521 (2)	0.2805 (13)	0.4354 (19)	0.060 (3)	0.234 (3)
S1	0.97554 (9)	0.45936 (7)	0.99949 (7)	0.0350 (2)	
S2	0.37194 (12)	0.62544 (6)	0.86134 (11)	0.0451 (3)	
S3	0.40982 (11)	0.25794 (6)	0.88610 (10)	0.0411 (3)	
C61	0.7135 (4)	0.4919 (2)	0.9390 (3)	0.0228 (7)	
C62	0.5760 (4)	0.5304 (2)	0.9076 (3)	0.0225 (7)	
C63	0.4502 (4)	0.4853 (2)	0.8857 (3)	0.0238 (7)	
C64	0.4586 (4)	0.4022 (2)	0.8906 (3)	0.0242 (7)	
C65	0.5932 (4)	0.3644 (2)	0.9175 (3)	0.0229 (7)	
C66	0.7225 (3)	0.4105 (2)	0.9436 (2)	0.0220 (7)	
C67	0.8582 (4)	0.3847 (2)	0.9754 (3)	0.0286 (8)	
H67	0.894 (4)	0.336 (3)	0.990 (4)	0.026 (11)*	
C68	0.8437 (4)	0.5261 (2)	0.9675 (3)	0.0296 (8)	
H68	0.861 (5)	0.582 (3)	0.964 (4)	0.035 (12)*	
C69	0.5487 (4)	0.6071 (2)	0.8962 (4)	0.0327 (8)	
H69	0.612 (5)	0.655 (3)	0.908 (4)	0.044 (14)*	
C70	0.3325 (4)	0.5305 (3)	0.8607 (4)	0.0364 (9)	
H70	0.227 (6)	0.521 (3)	0.847 (5)	0.054 (16)*	
C71	0.3499 (4)	0.3509 (2)	0.8712 (4)	0.0358 (9)	
H71	0.250 (5)	0.364 (3)	0.852 (4)	0.038 (13)*	
C72	0.5815 (4)	0.2864 (2)	0.9172 (3)	0.0315 (8)	
H72	0.651 (5)	0.249 (3)	0.930 (4)	0.041 (13)*	
C73	0.0949 (4)	0.6683 (2)	−0.0110 (4)	0.0386 (10)	
H73	0.178036	0.638756	−0.000314	0.046*	
C74	0.0235 (5)	0.6937 (3)	−0.1125 (4)	0.0437 (10)	
H74	0.057290	0.681793	−0.170883	0.052*	
C75	−0.0991 (5)	0.7371 (3)	−0.1315 (4)	0.0475 (11)	
H75	−0.149408	0.755155	−0.202192	0.057*	
C76	−0.1461 (4)	0.7533 (3)	−0.0441 (4)	0.0417 (10)	
H76	−0.229647	0.782528	−0.055457	0.050*	
C77	−0.0735 (4)	0.7275 (2)	0.0580 (4)	0.0379 (9)	
H77	−0.107738	0.739470	0.116135	0.045*	
C78	0.0505 (4)	0.6840 (2)	0.0789 (3)	0.0326 (8)	
C79	0.1281 (5)	0.6549 (3)	0.1891 (4)	0.0423 (10)	
H79A	0.079385	0.671085	0.239693	0.064*	
H79B	0.224224	0.675564	0.212767	0.064*	
H79C	0.131922	0.598718	0.187519	0.064*	

### Benzo[1,2-c:3,4-c':5,6-c'']trithiophene–\ buckminsterfullerene–toluene (II) Atomic displacement parameters (Å^2^)

**Table:**

	*U*^11^	*U*^22^	*U*^33^	*U*^12^	*U*^13^	*U*^23^
C1	0.071 (4)	0.037 (3)	0.047 (3)	0.030 (3)	0.010 (3)	−0.011 (3)
C2	0.058 (3)	0.043 (3)	0.045 (3)	0.033 (3)	0.014 (3)	0.004 (3)
C3	0.030 (2)	0.063 (3)	0.058 (3)	0.024 (2)	0.017 (2)	0.009 (3)
C4	0.031 (3)	0.062 (4)	0.054 (3)	0.008 (3)	0.026 (2)	−0.005 (3)
C5	0.046 (3)	0.067 (3)	0.039 (3)	0.014 (3)	0.029 (2)	−0.006 (3)
C6	0.064 (3)	0.051 (3)	0.041 (3)	0.017 (3)	0.023 (3)	−0.019 (3)
C7	0.066 (3)	0.035 (3)	0.051 (3)	0.029 (3)	0.013 (3)	0.016 (3)
C8	0.045 (3)	0.050 (3)	0.035 (3)	0.015 (3)	0.007 (2)	0.022 (2)
C9	0.031 (3)	0.065 (4)	0.037 (3)	0.004 (3)	−0.005 (2)	0.011 (3)
C10	0.020 (2)	0.060 (4)	0.052 (3)	0.006 (3)	0.002 (2)	0.018 (3)
C11	0.022 (2)	0.060 (3)	0.059 (3)	−0.013 (3)	0.007 (2)	−0.001 (3)
C12	0.023 (2)	0.065 (4)	0.067 (3)	−0.006 (3)	0.022 (2)	0.013 (3)
C13	0.040 (3)	0.062 (4)	0.066 (4)	−0.009 (3)	0.030 (3)	0.021 (3)
C14	0.052 (3)	0.066 (4)	0.045 (3)	−0.007 (3)	0.032 (3)	0.017 (3)
C15	0.063 (4)	0.047 (3)	0.040 (3)	−0.007 (3)	0.022 (3)	0.020 (3)
C16	0.079 (4)	0.032 (3)	0.055 (4)	−0.011 (3)	0.022 (3)	0.019 (3)
C17	0.059 (3)	0.046 (3)	0.068 (4)	−0.030 (3)	0.024 (3)	0.009 (3)
C18	0.061 (3)	0.041 (3)	0.067 (4)	−0.029 (3)	0.003 (3)	−0.004 (3)
C19	0.043 (3)	0.056 (4)	0.056 (3)	−0.025 (3)	−0.004 (3)	−0.008 (3)
C20	0.052 (4)	0.062 (3)	0.037 (3)	−0.015 (3)	−0.009 (3)	−0.020 (3)
C21	0.050 (3)	0.071 (4)	0.027 (3)	−0.006 (3)	−0.011 (3)	−0.003 (3)
C22	0.058 (3)	0.064 (4)	0.016 (2)	0.009 (3)	0.007 (2)	0.002 (2)
C23	0.076 (3)	0.066 (3)	0.022 (2)	0.018 (3)	0.020 (2)	−0.002 (2)
C24	0.069 (4)	0.057 (3)	0.032 (3)	0.019 (3)	0.012 (3)	−0.021 (2)
C25	0.073 (4)	0.048 (3)	0.041 (3)	−0.007 (3)	0.004 (3)	−0.025 (3)
C26	0.086 (4)	0.032 (3)	0.059 (4)	−0.018 (3)	0.013 (3)	−0.020 (3)
C27	0.099 (4)	0.020 (3)	0.057 (4)	0.006 (3)	0.007 (3)	−0.009 (3)
C28	0.080 (4)	0.043 (3)	0.051 (4)	0.037 (3)	0.016 (3)	−0.008 (3)
C29	0.075 (4)	0.062 (3)	0.049 (3)	0.029 (3)	0.029 (3)	−0.014 (3)
C30	0.061 (3)	0.076 (4)	0.044 (3)	0.029 (3)	0.036 (3)	0.004 (3)
C31	0.061 (3)	0.078 (4)	0.032 (3)	0.021 (3)	0.034 (2)	0.010 (3)
C32	0.057 (4)	0.080 (4)	0.042 (4)	−0.002 (3)	0.034 (3)	0.021 (3)
C33	0.036 (3)	0.077 (4)	0.064 (4)	−0.007 (3)	0.027 (3)	0.030 (3)
C34	0.024 (2)	0.076 (4)	0.067 (3)	0.003 (3)	0.026 (2)	0.023 (3)
C35	0.039 (3)	0.073 (4)	0.059 (3)	0.020 (3)	0.034 (3)	0.004 (3)
C36	0.081 (4)	0.025 (3)	0.047 (3)	0.002 (3)	0.019 (3)	0.012 (3)
C37	0.064 (4)	0.050 (3)	0.055 (4)	0.040 (3)	0.017 (3)	0.008 (3)
C38	0.069 (4)	0.035 (3)	0.049 (3)	0.031 (3)	0.018 (3)	0.016 (3)
C39	0.095 (4)	0.018 (2)	0.054 (4)	0.007 (3)	0.020 (3)	0.011 (3)
C40	0.055 (4)	0.048 (3)	0.026 (3)	0.006 (3)	0.012 (3)	0.019 (2)
C41	0.046 (4)	0.045 (3)	0.038 (3)	0.017 (3)	0.008 (3)	0.022 (3)
C42	0.039 (3)	0.067 (3)	0.061 (3)	0.033 (3)	0.023 (2)	0.016 (3)
C43	0.024 (2)	0.064 (4)	0.054 (3)	0.009 (3)	0.006 (2)	0.023 (3)
C44	0.019 (2)	0.067 (4)	0.063 (3)	−0.005 (3)	0.009 (2)	0.009 (3)
C45	0.033 (3)	0.057 (4)	0.058 (3)	−0.022 (3)	−0.006 (3)	−0.002 (3)
C46	0.055 (3)	0.046 (3)	0.063 (4)	−0.033 (3)	0.005 (3)	0.005 (3)
C47	0.042 (3)	0.059 (3)	0.038 (3)	−0.011 (3)	−0.010 (3)	−0.013 (3)
C48	0.043 (3)	0.066 (4)	0.026 (3)	−0.006 (3)	−0.008 (2)	0.000 (3)
C49	0.057 (3)	0.056 (3)	0.015 (2)	0.004 (3)	0.008 (2)	0.005 (2)
C50	0.064 (3)	0.062 (3)	0.020 (2)	0.009 (3)	0.020 (2)	−0.004 (2)
C51	0.057 (3)	0.064 (4)	0.034 (3)	0.006 (3)	0.033 (2)	0.004 (3)
C52	0.060 (3)	0.051 (3)	0.026 (3)	0.008 (3)	0.009 (3)	−0.022 (2)
C53	0.065 (4)	0.043 (3)	0.038 (3)	−0.015 (3)	0.005 (3)	−0.022 (2)
C54	0.074 (4)	0.030 (3)	0.058 (3)	−0.022 (3)	0.013 (3)	−0.016 (3)
C55	0.088 (4)	0.020 (3)	0.055 (4)	0.004 (3)	0.008 (3)	−0.005 (3)
C56	0.074 (4)	0.040 (3)	0.052 (4)	−0.014 (3)	0.020 (3)	0.021 (3)
C57	0.059 (4)	0.056 (4)	0.039 (3)	−0.006 (3)	0.020 (3)	0.022 (3)
C58	0.056 (3)	0.056 (3)	0.066 (4)	−0.028 (3)	0.022 (3)	0.015 (3)
C59	0.031 (3)	0.064 (4)	0.038 (3)	0.008 (3)	−0.004 (2)	0.019 (3)
C60	0.054 (4)	0.057 (3)	0.027 (3)	0.010 (3)	0.012 (3)	0.021 (2)
C1B	0.053 (5)	0.055 (5)	0.022 (4)	0.000 (5)	0.020 (4)	0.006 (4)
C2B	0.052 (6)	0.046 (5)	0.030 (5)	0.002 (5)	0.016 (5)	0.014 (5)
C3B	0.050 (6)	0.047 (5)	0.037 (5)	0.012 (5)	0.010 (5)	0.019 (5)
C4B	0.037 (5)	0.059 (6)	0.039 (5)	0.007 (5)	0.001 (5)	0.018 (5)
C5B	0.043 (5)	0.062 (5)	0.032 (5)	−0.003 (5)	−0.002 (5)	0.002 (5)
C6B	0.054 (5)	0.057 (5)	0.020 (4)	−0.002 (5)	0.009 (4)	−0.003 (5)
C7B	0.066 (5)	0.048 (5)	0.045 (5)	−0.016 (5)	0.018 (5)	0.017 (5)
C8B	0.077 (5)	0.037 (5)	0.055 (6)	−0.013 (5)	0.014 (5)	0.011 (5)
C9B	0.085 (5)	0.027 (5)	0.054 (6)	0.007 (5)	0.016 (5)	0.007 (5)
C10B	0.065 (5)	0.038 (5)	0.050 (5)	0.021 (5)	0.018 (5)	0.013 (5)
C11B	0.056 (5)	0.052 (5)	0.056 (5)	0.029 (5)	0.015 (5)	0.009 (5)
C12B	0.035 (5)	0.061 (5)	0.055 (5)	0.019 (5)	0.009 (5)	0.013 (5)
C13B	0.033 (5)	0.070 (5)	0.063 (5)	0.006 (5)	0.012 (4)	0.011 (5)
C14B	0.032 (5)	0.060 (5)	0.056 (5)	−0.011 (5)	0.005 (4)	0.000 (5)
C15B	0.042 (4)	0.065 (5)	0.064 (5)	−0.024 (4)	0.014 (4)	0.008 (5)
C16B	0.044 (5)	0.071 (5)	0.061 (5)	−0.008 (5)	0.029 (5)	0.018 (5)
C17B	0.035 (5)	0.068 (5)	0.057 (5)	0.011 (5)	0.028 (4)	0.006 (5)
C18B	0.051 (5)	0.070 (6)	0.054 (5)	0.025 (5)	0.030 (5)	−0.001 (5)
C19B	0.064 (5)	0.060 (5)	0.055 (5)	0.032 (4)	0.024 (5)	−0.001 (5)
C20B	0.077 (6)	0.045 (5)	0.053 (6)	0.025 (5)	0.016 (5)	−0.010 (5)
C21B	0.086 (6)	0.029 (5)	0.056 (6)	0.005 (5)	0.012 (5)	−0.007 (5)
C22B	0.072 (6)	0.039 (5)	0.059 (5)	−0.017 (5)	0.010 (5)	−0.011 (5)
C23B	0.061 (5)	0.049 (5)	0.047 (5)	−0.014 (5)	0.003 (5)	−0.019 (5)
C24B	0.063 (6)	0.058 (5)	0.035 (5)	0.000 (5)	0.012 (5)	−0.020 (5)
C25B	0.069 (5)	0.058 (5)	0.040 (5)	0.021 (5)	0.020 (5)	−0.020 (4)
C26B	0.066 (5)	0.070 (5)	0.040 (5)	0.020 (5)	0.030 (5)	−0.004 (5)
C27B	0.062 (5)	0.071 (6)	0.035 (5)	0.008 (5)	0.034 (5)	0.009 (5)
C28B	0.059 (5)	0.061 (5)	0.023 (4)	0.007 (5)	0.019 (4)	0.009 (4)
C29B	0.058 (5)	0.060 (5)	0.020 (4)	0.004 (5)	0.008 (4)	−0.007 (5)
C30B	0.047 (5)	0.064 (5)	0.030 (5)	−0.002 (5)	−0.003 (5)	−0.004 (5)
C31B	0.045 (5)	0.060 (5)	0.045 (5)	−0.015 (5)	0.001 (5)	−0.012 (5)
C32B	0.032 (5)	0.057 (5)	0.057 (5)	−0.014 (5)	0.007 (4)	−0.004 (5)
C33B	0.026 (4)	0.064 (5)	0.061 (5)	0.002 (5)	0.013 (4)	0.007 (5)
C34B	0.032 (5)	0.059 (5)	0.053 (5)	0.012 (5)	0.007 (5)	0.011 (5)
C35B	0.039 (6)	0.060 (5)	0.037 (5)	0.003 (5)	−0.001 (5)	0.016 (5)
C36B	0.054 (5)	0.059 (5)	0.047 (5)	−0.013 (5)	0.028 (5)	0.014 (5)
C37B	0.053 (6)	0.049 (5)	0.029 (5)	0.001 (5)	0.015 (5)	0.018 (5)
C38B	0.064 (5)	0.055 (5)	0.044 (5)	−0.016 (5)	0.019 (5)	0.021 (5)
C39B	0.052 (5)	0.068 (5)	0.046 (5)	−0.015 (5)	0.030 (5)	0.016 (5)
C40B	0.067 (5)	0.045 (5)	0.059 (6)	−0.028 (5)	0.013 (5)	0.008 (5)
C41B	0.072 (5)	0.044 (5)	0.053 (6)	−0.014 (5)	0.011 (5)	0.017 (5)
C42B	0.050 (6)	0.050 (5)	0.038 (5)	0.011 (5)	0.006 (5)	0.018 (5)
C43B	0.062 (5)	0.040 (5)	0.049 (5)	0.019 (5)	0.013 (5)	0.012 (5)
C44B	0.047 (5)	0.053 (5)	0.057 (6)	0.029 (5)	0.010 (5)	0.008 (5)
C45B	0.058 (5)	0.059 (5)	0.052 (5)	0.023 (4)	0.020 (4)	−0.003 (5)
C46B	0.045 (5)	0.066 (5)	0.050 (5)	0.015 (5)	0.028 (5)	−0.007 (5)
C47B	0.072 (6)	0.039 (5)	0.048 (6)	0.020 (5)	0.011 (5)	−0.011 (5)
C48B	0.077 (6)	0.029 (5)	0.049 (6)	0.002 (5)	0.012 (5)	−0.006 (5)
C49B	0.066 (6)	0.033 (5)	0.057 (5)	−0.020 (5)	0.008 (5)	−0.010 (5)
C50B	0.055 (5)	0.045 (5)	0.048 (5)	−0.017 (5)	0.002 (5)	−0.017 (5)
C51B	0.041 (5)	0.059 (5)	0.043 (5)	−0.014 (5)	−0.001 (5)	−0.012 (5)
C52B	0.058 (6)	0.049 (5)	0.032 (5)	−0.004 (5)	0.010 (5)	−0.019 (5)
C53B	0.061 (5)	0.054 (5)	0.039 (5)	0.013 (5)	0.016 (5)	−0.019 (4)
C54B	0.057 (5)	0.062 (5)	0.037 (5)	0.013 (5)	0.026 (4)	−0.010 (5)
C55B	0.052 (5)	0.063 (6)	0.031 (5)	0.003 (5)	0.031 (5)	0.003 (5)
C56B	0.044 (5)	0.063 (6)	0.059 (5)	−0.012 (5)	0.028 (5)	0.013 (5)
C57B	0.046 (5)	0.061 (5)	0.064 (5)	−0.025 (4)	0.015 (4)	0.003 (5)
C58B	0.029 (4)	0.063 (5)	0.055 (5)	0.004 (5)	0.025 (4)	0.003 (5)
C59B	0.074 (5)	0.030 (5)	0.050 (6)	0.007 (5)	0.016 (5)	0.007 (5)
C60B	0.070 (5)	0.046 (5)	0.061 (6)	−0.028 (5)	0.013 (5)	0.003 (5)
S1	0.0235 (4)	0.0519 (6)	0.0304 (4)	−0.0039 (4)	0.0095 (3)	−0.0032 (4)
S2	0.0432 (5)	0.0275 (5)	0.0670 (7)	0.0106 (4)	0.0213 (5)	0.0104 (5)
S3	0.0426 (5)	0.0246 (5)	0.0532 (6)	−0.0103 (4)	0.0115 (4)	−0.0030 (4)
C61	0.0261 (16)	0.0251 (16)	0.0179 (16)	−0.0032 (13)	0.0081 (13)	−0.0018 (13)
C62	0.0287 (16)	0.0213 (17)	0.0182 (16)	−0.0002 (14)	0.0086 (13)	−0.0005 (12)
C63	0.0254 (16)	0.0247 (17)	0.0198 (16)	−0.0017 (14)	0.0053 (13)	0.0018 (13)
C64	0.0278 (16)	0.0239 (18)	0.0179 (16)	−0.0034 (13)	0.0033 (13)	0.0006 (12)
C65	0.0264 (16)	0.0252 (17)	0.0176 (16)	−0.0006 (14)	0.0079 (13)	−0.0010 (13)
C66	0.0265 (16)	0.0285 (17)	0.0133 (15)	−0.0008 (14)	0.0096 (13)	−0.0008 (13)
C67	0.0280 (17)	0.035 (2)	0.0244 (18)	0.0033 (16)	0.0110 (14)	0.0007 (15)
C68	0.0318 (18)	0.034 (2)	0.0248 (19)	−0.0083 (16)	0.0122 (15)	−0.0070 (15)
C69	0.0385 (19)	0.0244 (19)	0.039 (2)	−0.0003 (16)	0.0179 (17)	0.0004 (15)
C70	0.0277 (18)	0.034 (2)	0.044 (2)	0.0042 (17)	0.0067 (17)	0.0064 (18)
C71	0.0282 (18)	0.033 (2)	0.040 (2)	−0.0064 (16)	0.0029 (17)	0.0040 (17)
C72	0.0369 (19)	0.0256 (18)	0.035 (2)	−0.0010 (16)	0.0156 (16)	−0.0027 (15)
C73	0.0321 (19)	0.032 (2)	0.059 (3)	−0.0077 (16)	0.0244 (19)	−0.0094 (19)
C74	0.044 (2)	0.046 (3)	0.045 (2)	−0.013 (2)	0.018 (2)	−0.0103 (19)
C75	0.043 (2)	0.046 (3)	0.050 (3)	−0.014 (2)	0.010 (2)	−0.003 (2)
C76	0.0291 (18)	0.038 (2)	0.054 (3)	−0.0022 (18)	0.0079 (17)	0.003 (2)
C77	0.0295 (18)	0.0284 (19)	0.060 (3)	−0.0018 (15)	0.0208 (19)	−0.0044 (18)
C78	0.0260 (17)	0.0275 (19)	0.043 (2)	−0.0051 (15)	0.0099 (16)	−0.0047 (16)
C79	0.042 (2)	0.035 (2)	0.050 (3)	−0.0001 (19)	0.0151 (19)	0.0013 (19)

### Benzo[1,2-c:3,4-c':5,6-c'']trithiophene–\ buckminsterfullerene–toluene (II) Geometric parameters (Å, º)

**Table:**

C1—C6	1.402 (11)	C11B—C12B	1.45 (2)
C1—C2	1.415 (11)	C12B—C13B	1.37 (2)
C1—C55	1.443 (13)	C13B—C17B	1.43 (2)
C2—C3	1.379 (11)	C13B—C14B	1.46 (2)
C2—C7	1.456 (13)	C14B—C51B	1.39 (2)
C3—C10	1.435 (11)	C14B—C15B	1.48 (2)
C3—C4	1.461 (10)	C15B—C40B	1.37 (2)
C4—C5	1.380 (11)	C15B—C16B	1.44 (2)
C4—C12	1.455 (11)	C16B—C39B	1.36 (2)
C5—C51	1.422 (12)	C16B—C17B	1.48 (2)
C5—C6	1.430 (11)	C17B—C18B	1.38 (2)
C6—C52	1.474 (10)	C18B—C19B	1.41 (2)
C7—C8	1.384 (12)	C18B—C26B	1.54 (2)
C7—C36	1.479 (13)	C19B—C20B	1.48 (2)
C8—C60	1.424 (11)	C20B—C21B	1.39 (3)
C8—C9	1.447 (11)	C20B—C25B	1.49 (2)
C9—C10	1.391 (12)	C21B—C22B	1.44 (3)
C9—C21	1.442 (13)	C22B—C23B	1.36 (2)
C10—C11	1.431 (10)	C22B—C60B	1.40 (3)
C11—C19	1.373 (11)	C23B—C31B	1.44 (2)
C11—C12	1.476 (11)	C23B—C24B	1.48 (2)
C12—C13	1.377 (11)	C24B—C25B	1.32 (2)
C13—C17	1.414 (12)	C24B—C29B	1.46 (2)
C13—C14	1.458 (12)	C25B—C26B	1.44 (2)
C14—C51	1.386 (13)	C26B—C27B	1.44 (2)
C14—C15	1.479 (11)	C27B—C28B	1.44 (3)
C15—C40	1.368 (11)	C27B—C39B	1.45 (3)
C15—C16	1.437 (12)	C28B—C29B	1.39 (2)
C16—C39	1.364 (13)	C28B—C37B	1.41 (2)
C16—C17	1.489 (12)	C29B—C30B	1.46 (3)
C17—C18	1.366 (11)	C30B—C35B	1.37 (3)
C18—C19	1.418 (12)	C30B—C31B	1.44 (3)
C18—C26	1.517 (13)	C31B—C32B	1.33 (2)
C19—C20	1.469 (12)	C32B—C33B	1.45 (2)
C20—C21	1.398 (14)	C32B—C57B	1.46 (2)
C20—C25	1.487 (12)	C33B—C34B	1.39 (2)
C21—C22	1.447 (13)	C33B—C58B	1.43 (2)
C22—C23	1.373 (10)	C34B—C35B	1.44 (3)
C22—C60	1.436 (12)	C34B—C44B	1.47 (2)
C23—C31	1.446 (12)	C35B—C42B	1.47 (3)
C23—C24	1.465 (12)	C36B—C56B	1.36 (3)
C24—C25	1.341 (13)	C36B—C55B	1.46 (2)
C24—C29	1.463 (11)	C37B—C42B	1.40 (3)
C25—C26	1.457 (13)	C37B—C38B	1.42 (2)
C26—C27	1.400 (13)	C38B—C41B	1.41 (2)
C27—C28	1.440 (14)	C38B—C39B	1.49 (3)
C27—C39	1.466 (12)	C40B—C49B	1.42 (3)
C28—C29	1.385 (12)	C40B—C41B	1.45 (2)
C28—C37	1.419 (12)	C41B—C59B	1.43 (3)
C29—C30	1.450 (13)	C42B—C43B	1.44 (3)
C30—C35	1.368 (13)	C43B—C59B	1.38 (3)
C30—C31	1.460 (12)	C43B—C44B	1.44 (3)
C31—C32	1.347 (13)	C44B—C45B	1.42 (2)
C32—C33	1.462 (12)	C45B—C46B	1.44 (2)
C32—C57	1.469 (11)	C45B—C47B	1.50 (2)
C33—C58	1.406 (13)	C46B—C58B	1.37 (2)
C33—C34	1.407 (11)	C46B—C54B	1.53 (2)
C34—C35	1.443 (12)	C47B—C48B	1.38 (3)
C34—C44	1.478 (11)	C47B—C53B	1.49 (2)
C35—C42	1.467 (11)	C48B—C59B	1.46 (3)
C36—C56	1.352 (13)	C48B—C49B	1.47 (3)
C36—C55	1.465 (12)	C49B—C50B	1.38 (2)
C37—C42	1.411 (12)	C50B—C51B	1.43 (2)
C37—C38	1.448 (13)	C50B—C52B	1.46 (2)
C38—C41	1.397 (12)	C52B—C53B	1.34 (2)
C38—C39	1.497 (13)	C53B—C54B	1.45 (2)
C40—C49	1.437 (11)	C54B—C55B	1.41 (2)
C40—C41	1.439 (11)	C56B—C57B	1.43 (2)
C41—C59	1.451 (11)	C56B—C58B	1.45 (2)
C42—C43	1.437 (12)	C57B—C60B	1.40 (2)
C43—C59	1.382 (11)	S1—C68	1.707 (4)
C43—C44	1.429 (10)	S1—C67	1.713 (4)
C44—C45	1.398 (11)	S2—C70	1.701 (5)
C45—C46	1.441 (11)	S2—C69	1.710 (4)
C45—C47	1.473 (12)	S3—C72	1.707 (4)
C46—C58	1.370 (11)	S3—C71	1.717 (5)
C46—C54	1.522 (12)	C61—C68	1.371 (5)
C47—C48	1.378 (13)	C61—C66	1.422 (5)
C47—C53	1.465 (11)	C61—C62	1.466 (5)
C48—C59	1.448 (13)	C62—C69	1.363 (5)
C48—C49	1.464 (12)	C62—C63	1.434 (5)
C49—C50	1.373 (9)	C63—C70	1.366 (5)
C50—C51	1.430 (11)	C63—C64	1.451 (5)
C50—C52	1.443 (10)	C64—C71	1.368 (5)
C52—C53	1.367 (12)	C64—C65	1.438 (5)
C53—C54	1.430 (12)	C65—C72	1.364 (5)
C54—C55	1.392 (12)	C65—C66	1.469 (5)
C56—C57	1.455 (12)	C66—C67	1.363 (5)
C56—C58	1.476 (12)	C67—H67	0.92 (5)
C57—C60	1.385 (11)	C68—H68	0.99 (5)
C1B—C2B	1.41 (2)	C69—H69	1.03 (6)
C1B—C6B	1.42 (2)	C70—H70	1.03 (6)
C1B—C55B	1.44 (2)	C71—H71	0.98 (5)
C2B—C3B	1.38 (3)	C72—H72	0.92 (5)
C2B—C7B	1.44 (2)	C73—C74	1.359 (7)
C3B—C10B	1.43 (3)	C73—C78	1.407 (6)
C3B—C4B	1.46 (2)	C73—H73	0.9500
C4B—C5B	1.39 (3)	C74—C75	1.395 (7)
C4B—C12B	1.45 (3)	C74—H74	0.9500
C5B—C51B	1.42 (3)	C75—C76	1.394 (7)
C5B—C6B	1.46 (3)	C75—H75	0.9500
C6B—C52B	1.47 (2)	C76—C77	1.369 (7)
C7B—C8B	1.38 (2)	C76—H76	0.9500
C7B—C36B	1.46 (3)	C77—C78	1.405 (6)
C8B—C60B	1.42 (2)	C77—H77	0.9500
C8B—C9B	1.44 (3)	C78—C79	1.484 (6)
C9B—C10B	1.38 (3)	C79—H79A	0.9800
C9B—C21B	1.45 (3)	C79—H79B	0.9800
C10B—C11B	1.46 (3)	C79—H79C	0.9800
C11B—C19B	1.39 (2)		
			
C6—C1—C2	120.5 (8)	C12B—C11B—C10B	109 (2)
C6—C1—C55	120.0 (7)	C13B—C12B—C11B	121 (2)
C2—C1—C55	107.9 (7)	C13B—C12B—C4B	122 (2)
C3—C2—C1	121.2 (7)	C11B—C12B—C4B	107.0 (19)
C3—C2—C7	118.4 (8)	C12B—C13B—C17B	117 (2)
C1—C2—C7	108.5 (8)	C12B—C13B—C14B	118.0 (19)
C2—C3—C10	121.5 (7)	C17B—C13B—C14B	111.9 (19)
C2—C3—C4	118.1 (7)	C51B—C14B—C13B	120 (2)
C10—C3—C4	108.4 (7)	C51B—C14B—C15B	124 (2)
C5—C4—C12	118.8 (7)	C13B—C14B—C15B	103.7 (18)
C5—C4—C3	121.0 (8)	C40B—C15B—C16B	116.5 (19)
C12—C4—C3	107.8 (7)	C40B—C15B—C14B	120.7 (19)
C4—C5—C51	121.2 (8)	C16B—C15B—C14B	110.4 (18)
C4—C5—C6	119.7 (7)	C39B—C16B—C15B	117 (2)
C51—C5—C6	107.1 (7)	C39B—C16B—C17B	125 (2)
C1—C6—C5	119.4 (7)	C15B—C16B—C17B	107.1 (18)
C1—C6—C52	120.1 (7)	C18B—C17B—C13B	122 (2)
C5—C6—C52	108.5 (7)	C18B—C17B—C16B	121.7 (19)
C8—C7—C2	121.0 (10)	C13B—C17B—C16B	106.7 (19)
C8—C7—C36	118.1 (10)	C17B—C18B—C19B	123 (2)
C2—C7—C36	109.2 (9)	C17B—C18B—C26B	116.5 (19)
C7—C8—C60	119.9 (8)	C19B—C18B—C26B	107.0 (18)
C7—C8—C9	119.4 (8)	C11B—C19B—C18B	115 (2)
C60—C8—C9	108.9 (8)	C11B—C19B—C20B	119 (2)
C10—C9—C21	120.6 (10)	C18B—C19B—C20B	112.6 (18)
C10—C9—C8	120.0 (8)	C21B—C20B—C19B	126 (2)
C21—C9—C8	106.8 (8)	C21B—C20B—C25B	119 (2)
C9—C10—C11	120.2 (8)	C19B—C20B—C25B	103.3 (18)
C9—C10—C3	119.7 (7)	C20B—C21B—C22B	125 (3)
C11—C10—C3	108.5 (7)	C20B—C21B—C9B	111 (2)
C19—C11—C10	120.8 (7)	C22B—C21B—C9B	111 (2)
C19—C11—C12	118.6 (7)	C23B—C22B—C60B	130 (3)
C10—C11—C12	108.5 (7)	C23B—C22B—C21B	113 (2)
C13—C12—C4	121.7 (7)	C60B—C22B—C21B	105 (2)
C13—C12—C11	119.8 (8)	C22B—C23B—C31B	114 (2)
C4—C12—C11	106.7 (7)	C22B—C23B—C24B	125 (2)
C12—C13—C17	119.6 (8)	C31B—C23B—C24B	107.7 (18)
C12—C13—C14	117.5 (8)	C25B—C24B—C29B	120 (2)
C17—C13—C14	110.4 (7)	C25B—C24B—C23B	121 (2)
C51—C14—C13	121.5 (8)	C29B—C24B—C23B	107.8 (17)
C51—C14—C15	120.5 (7)	C24B—C25B—C26B	119 (2)
C13—C14—C15	106.2 (7)	C24B—C25B—C20B	117 (2)
C40—C15—C16	119.7 (8)	C26B—C25B—C20B	112.1 (19)
C40—C15—C14	119.7 (8)	C25B—C26B—C27B	124 (2)
C16—C15—C14	108.3 (7)	C25B—C26B—C18B	104.9 (18)
C39—C16—C15	119.8 (9)	C27B—C26B—C18B	117 (2)
C39—C16—C17	119.8 (8)	C28B—C27B—C26B	115 (2)
C15—C16—C17	107.8 (8)	C28B—C27B—C39B	111 (2)
C18—C17—C13	120.9 (8)	C26B—C27B—C39B	125 (2)
C18—C17—C16	120.7 (8)	C29B—C28B—C37B	121.8 (18)
C13—C17—C16	107.2 (7)	C29B—C28B—C27B	119.6 (18)
C17—C18—C19	120.8 (8)	C37B—C28B—C27B	108.1 (18)
C17—C18—C26	118.8 (8)	C28B—C29B—C24B	122.4 (18)
C19—C18—C26	108.9 (8)	C28B—C29B—C30B	117 (2)
C11—C19—C18	120.3 (8)	C24B—C29B—C30B	106.7 (19)
C11—C19—C20	119.2 (8)	C35B—C30B—C31B	118 (3)
C18—C19—C20	108.5 (8)	C35B—C30B—C29B	121 (3)
C21—C20—C19	120.4 (9)	C31B—C30B—C29B	109 (2)
C21—C20—C25	118.0 (8)	C32B—C31B—C23B	119 (2)
C19—C20—C25	108.3 (8)	C32B—C31B—C30B	122 (2)
C20—C21—C9	118.7 (10)	C23B—C31B—C30B	109 (2)
C20—C21—C22	122.1 (10)	C31B—C32B—C33B	123 (2)
C9—C21—C22	108.4 (9)	C31B—C32B—C57B	125 (2)
C23—C22—C60	121.4 (7)	C33B—C32B—C57B	100.9 (18)
C23—C22—C21	118.7 (8)	C34B—C33B—C58B	120 (2)
C60—C22—C21	107.6 (8)	C34B—C33B—C32B	114.8 (18)
C22—C23—C31	120.4 (7)	C58B—C33B—C32B	113.4 (19)
C22—C23—C24	119.8 (7)	C33B—C34B—C35B	123 (2)
C31—C23—C24	108.3 (7)	C33B—C34B—C44B	118 (2)
C25—C24—C29	118.7 (8)	C35B—C34B—C44B	108.0 (19)
C25—C24—C23	122.4 (8)	C30B—C35B—C34B	120 (3)
C29—C24—C23	107.7 (7)	C30B—C35B—C42B	122 (3)
C24—C25—C26	121.3 (8)	C34B—C35B—C42B	106 (2)
C24—C25—C20	119.1 (8)	C56B—C36B—C55B	117 (2)
C26—C25—C20	107.6 (8)	C56B—C36B—C7B	125 (2)
C27—C26—C25	120.1 (9)	C55B—C36B—C7B	103.2 (19)
C27—C26—C18	120.1 (9)	C42B—C37B—C28B	122 (2)
C25—C26—C18	106.7 (8)	C42B—C37B—C38B	116 (2)
C26—C27—C28	118.8 (9)	C28B—C37B—C38B	108.3 (19)
C26—C27—C39	118.7 (10)	C41B—C38B—C37B	125 (2)
C28—C27—C39	111.1 (9)	C41B—C38B—C39B	115 (2)
C29—C28—C37	122.2 (9)	C37B—C38B—C39B	109.8 (19)
C29—C28—C27	119.7 (8)	C16B—C39B—C27B	115 (2)
C37—C28—C27	106.2 (8)	C16B—C39B—C38B	127 (2)
C28—C29—C30	118.1 (8)	C27B—C39B—C38B	103.1 (19)
C28—C29—C24	121.2 (8)	C15B—C40B—C49B	112 (2)
C30—C29—C24	107.6 (7)	C15B—C40B—C41B	128 (2)
C35—C30—C29	120.5 (8)	C49B—C40B—C41B	107.1 (19)
C35—C30—C31	119.4 (8)	C38B—C41B—C59B	118 (2)
C29—C30—C31	108.7 (8)	C38B—C41B—C40B	116 (2)
C32—C31—C23	119.4 (8)	C59B—C41B—C40B	113 (2)
C32—C31—C30	120.3 (9)	C37B—C42B—C43B	120 (3)
C23—C31—C30	107.7 (8)	C37B—C42B—C35B	116 (2)
C31—C32—C33	122.8 (8)	C43B—C42B—C35B	111 (2)
C31—C32—C57	120.4 (8)	C59B—C43B—C42B	123 (3)
C33—C32—C57	105.8 (8)	C59B—C43B—C44B	120 (3)
C58—C33—C34	120.9 (8)	C42B—C43B—C44B	106 (2)
C58—C33—C32	111.0 (7)	C45B—C44B—C43B	117 (2)
C34—C33—C32	115.4 (8)	C45B—C44B—C34B	122.5 (19)
C33—C34—C35	122.5 (7)	C43B—C44B—C34B	110 (2)
C33—C34—C44	117.9 (8)	C44B—C45B—C46B	116.1 (19)
C35—C34—C44	107.1 (7)	C44B—C45B—C47B	117.7 (19)
C30—C35—C34	119.6 (7)	C46B—C45B—C47B	111.1 (18)
C30—C35—C42	121.9 (9)	C58B—C46B—C45B	122 (2)
C34—C35—C42	107.0 (8)	C58B—C46B—C54B	118.9 (19)
C56—C36—C55	121.5 (9)	C45B—C46B—C54B	107.6 (17)
C56—C36—C7	121.7 (10)	C48B—C47B—C53B	119 (2)
C55—C36—C7	103.9 (9)	C48B—C47B—C45B	128 (2)
C42—C37—C28	120.7 (8)	C53B—C47B—C45B	103.2 (18)
C42—C37—C38	116.6 (8)	C47B—C48B—C59B	109 (2)
C28—C37—C38	111.2 (9)	C47B—C48B—C49B	124 (3)
C41—C38—C37	122.5 (10)	C59B—C48B—C49B	112 (2)
C41—C38—C39	118.3 (9)	C50B—C49B—C40B	130 (2)
C37—C38—C39	106.8 (9)	C50B—C49B—C48B	114 (2)
C16—C39—C27	121.8 (10)	C40B—C49B—C48B	105.6 (19)
C16—C39—C38	120.9 (9)	C49B—C50B—C51B	118 (2)
C27—C39—C38	104.6 (9)	C49B—C50B—C52B	123 (2)
C15—C40—C49	118.7 (7)	C51B—C50B—C52B	108.0 (17)
C15—C40—C41	122.8 (8)	C14B—C51B—C5B	120 (2)
C49—C40—C41	107.1 (7)	C14B—C51B—C50B	115 (2)
C38—C41—C40	118.4 (8)	C5B—C51B—C50B	110 (2)
C38—C41—C59	119.3 (9)	C53B—C52B—C50B	122 (2)
C40—C41—C59	109.6 (8)	C53B—C52B—C6B	121 (2)
C37—C42—C43	121.1 (7)	C50B—C52B—C6B	106.3 (16)
C37—C42—C35	116.6 (7)	C52B—C53B—C54B	117 (2)
C43—C42—C35	109.5 (7)	C52B—C53B—C47B	117.4 (19)
C59—C43—C44	119.2 (8)	C54B—C53B—C47B	113.1 (19)
C59—C43—C42	121.4 (7)	C55B—C54B—C53B	125 (2)
C44—C43—C42	107.2 (7)	C55B—C54B—C46B	116.1 (19)
C45—C44—C43	120.0 (7)	C53B—C54B—C46B	104.7 (17)
C45—C44—C34	119.7 (7)	C54B—C55B—C1B	117 (2)
C43—C44—C34	109.2 (8)	C54B—C55B—C36B	124 (2)
C44—C45—C46	119.8 (8)	C1B—C55B—C36B	108.4 (18)
C44—C45—C47	120.0 (7)	C36B—C56B—C57B	119 (2)
C46—C45—C47	107.3 (7)	C36B—C56B—C58B	124 (2)
C58—C46—C45	120.0 (8)	C57B—C56B—C58B	106.3 (19)
C58—C46—C54	119.9 (7)	C60B—C57B—C56B	115.9 (19)
C45—C46—C54	108.3 (7)	C60B—C57B—C32B	117.6 (19)
C48—C47—C53	119.6 (8)	C56B—C57B—C32B	113.0 (18)
C48—C47—C45	119.9 (8)	C46B—C58B—C33B	122 (2)
C53—C47—C45	108.6 (7)	C46B—C58B—C56B	120.9 (19)
C47—C48—C59	118.4 (9)	C33B—C58B—C56B	106.2 (19)
C47—C48—C49	121.8 (9)	C43B—C59B—C41B	118 (3)
C59—C48—C49	107.4 (9)	C43B—C59B—C48B	128 (3)
C50—C49—C40	121.9 (7)	C41B—C59B—C48B	102 (2)
C50—C49—C48	117.8 (8)	C22B—C60B—C57B	114 (2)
C40—C49—C48	108.8 (7)	C22B—C60B—C8B	111 (2)
C49—C50—C51	120.5 (7)	C57B—C60B—C8B	124 (2)
C49—C50—C52	120.3 (6)	C68—S1—C67	92.40 (18)
C51—C50—C52	107.5 (6)	C70—S2—C69	92.1 (2)
C14—C51—C5	119.3 (8)	C72—S3—C71	92.4 (2)
C14—C51—C50	118.6 (8)	C68—C61—C66	112.3 (3)
C5—C51—C50	110.2 (8)	C68—C61—C62	127.0 (4)
C53—C52—C50	122.7 (7)	C66—C61—C62	120.6 (3)
C53—C52—C6	118.6 (7)	C69—C62—C63	112.6 (3)
C50—C52—C6	106.6 (6)	C69—C62—C61	128.0 (3)
C52—C53—C54	121.0 (7)	C63—C62—C61	119.4 (3)
C52—C53—C47	117.8 (8)	C70—C63—C62	111.5 (3)
C54—C53—C47	109.0 (7)	C70—C63—C64	128.3 (4)
C55—C54—C53	121.6 (9)	C62—C63—C64	120.2 (3)
C55—C54—C46	119.0 (8)	C71—C64—C65	111.8 (4)
C53—C54—C46	106.7 (7)	C71—C64—C63	127.8 (4)
C54—C55—C1	118.7 (9)	C65—C64—C63	120.3 (3)
C54—C55—C36	119.2 (10)	C72—C65—C64	112.5 (3)
C1—C55—C36	110.6 (8)	C72—C65—C66	127.9 (3)
C36—C56—C57	119.5 (8)	C64—C65—C66	119.5 (3)
C36—C56—C58	121.1 (8)	C67—C66—C61	112.7 (3)
C57—C56—C58	107.9 (8)	C67—C66—C65	127.5 (4)
C60—C57—C56	118.9 (8)	C61—C66—C65	119.8 (3)
C60—C57—C32	120.4 (8)	C66—C67—S1	111.3 (3)
C56—C57—C32	108.0 (7)	C66—C67—H67	131 (3)
C46—C58—C33	121.6 (8)	S1—C67—H67	118 (3)
C46—C58—C56	119.3 (8)	C61—C68—S1	111.3 (3)
C33—C58—C56	107.1 (8)	C61—C68—H68	125 (3)
C43—C59—C48	122.6 (8)	S1—C68—H68	123 (3)
C43—C59—C41	118.9 (8)	C62—C69—S2	111.5 (3)
C48—C59—C41	107.1 (8)	C62—C69—H69	134 (3)
C57—C60—C8	121.9 (8)	S2—C69—H69	115 (3)
C57—C60—C22	118.0 (7)	C63—C70—S2	112.3 (3)
C8—C60—C22	108.3 (7)	C63—C70—H70	135 (3)
C2B—C1B—C6B	118.7 (18)	S2—C70—H70	113 (3)
C2B—C1B—C55B	111.5 (18)	C64—C71—S3	111.6 (3)
C6B—C1B—C55B	117.9 (17)	C64—C71—H71	125 (3)
C3B—C2B—C1B	126 (2)	S3—C71—H71	123 (3)
C3B—C2B—C7B	117 (2)	C65—C72—S3	111.6 (3)
C1B—C2B—C7B	104.3 (19)	C65—C72—H72	130 (3)
C2B—C3B—C10B	123 (3)	S3—C72—H72	119 (3)
C2B—C3B—C4B	116 (2)	C74—C73—C78	122.6 (4)
C10B—C3B—C4B	109 (2)	C74—C73—H73	118.7
C5B—C4B—C12B	119 (2)	C78—C73—H73	118.7
C5B—C4B—C3B	118 (3)	C73—C74—C75	120.4 (5)
C12B—C4B—C3B	108 (2)	C73—C74—H74	119.8
C4B—C5B—C51B	120 (3)	C75—C74—H74	119.8
C4B—C5B—C6B	125 (3)	C76—C75—C74	118.3 (5)
C51B—C5B—C6B	107 (2)	C76—C75—H75	120.8
C1B—C6B—C5B	116 (2)	C74—C75—H75	120.8
C1B—C6B—C52B	122.0 (17)	C77—C76—C75	120.9 (4)
C5B—C6B—C52B	108.5 (18)	C77—C76—H76	119.5
C8B—C7B—C2B	120 (2)	C75—C76—H76	119.5
C8B—C7B—C36B	115 (2)	C76—C77—C78	121.7 (4)
C2B—C7B—C36B	113 (2)	C76—C77—H77	119.2
C7B—C8B—C60B	120 (2)	C78—C77—H77	119.2
C7B—C8B—C9B	122 (2)	C77—C78—C73	116.1 (4)
C60B—C8B—C9B	108 (2)	C77—C78—C79	121.8 (4)
C10B—C9B—C8B	119 (3)	C73—C78—C79	122.1 (4)
C10B—C9B—C21B	125 (3)	C78—C79—H79A	109.5
C8B—C9B—C21B	105 (2)	C78—C79—H79B	109.5
C9B—C10B—C3B	119 (3)	H79A—C79—H79B	109.5
C9B—C10B—C11B	122 (3)	C78—C79—H79C	109.5
C3B—C10B—C11B	107 (2)	H79A—C79—H79C	109.5
C19B—C11B—C12B	122 (2)	H79B—C79—H79C	109.5
C19B—C11B—C10B	116 (2)		
			
C6—C1—C2—C3	−0.4 (10)	C1B—C2B—C7B—C8B	142 (3)
C55—C1—C2—C3	142.7 (8)	C3B—C2B—C7B—C36B	−144 (3)
C6—C1—C2—C7	−142.4 (9)	C1B—C2B—C7B—C36B	1 (3)
C55—C1—C2—C7	0.6 (11)	C2B—C7B—C8B—C60B	−139 (3)
C1—C2—C3—C10	−137.1 (7)	C36B—C7B—C8B—C60B	0 (5)
C7—C2—C3—C10	1.3 (11)	C2B—C7B—C8B—C9B	3 (5)
C1—C2—C3—C4	1.2 (9)	C36B—C7B—C8B—C9B	142 (3)
C7—C2—C3—C4	139.6 (9)	C7B—C8B—C9B—C10B	−2 (6)
C2—C3—C4—C5	−1.5 (9)	C60B—C8B—C9B—C10B	144 (4)
C10—C3—C4—C5	141.8 (7)	C7B—C8B—C9B—C21B	−147 (4)
C2—C3—C4—C12	−143.0 (6)	C60B—C8B—C9B—C21B	−1 (4)
C10—C3—C4—C12	0.3 (7)	C8B—C9B—C10B—C3B	2 (7)
C12—C4—C5—C51	0.5 (10)	C21B—C9B—C10B—C3B	139 (4)
C3—C4—C5—C51	−137.1 (8)	C8B—C9B—C10B—C11B	−137 (4)
C12—C4—C5—C6	138.4 (7)	C21B—C9B—C10B—C11B	0 (8)
C3—C4—C5—C6	0.9 (10)	C2B—C3B—C10B—C9B	−2 (6)
C2—C1—C6—C5	−0.2 (10)	C4B—C3B—C10B—C9B	−143 (4)
C55—C1—C6—C5	−138.9 (8)	C2B—C3B—C10B—C11B	142 (3)
C2—C1—C6—C52	138.2 (7)	C4B—C3B—C10B—C11B	1 (5)
C55—C1—C6—C52	−0.5 (11)	C9B—C10B—C11B—C19B	−2 (6)
C4—C5—C6—C1	0.0 (9)	C3B—C10B—C11B—C19B	−145 (3)
C51—C5—C6—C1	143.1 (7)	C9B—C10B—C11B—C12B	140 (4)
C4—C5—C6—C52	−142.8 (6)	C3B—C10B—C11B—C12B	−3 (4)
C51—C5—C6—C52	0.4 (7)	C19B—C11B—C12B—C13B	−3 (4)
C3—C2—C7—C8	−1.0 (17)	C10B—C11B—C12B—C13B	−143 (3)
C1—C2—C7—C8	142.3 (11)	C19B—C11B—C12B—C4B	143 (3)
C3—C2—C7—C36	−143.3 (9)	C10B—C11B—C12B—C4B	4 (4)
C1—C2—C7—C36	0.0 (14)	C5B—C4B—C12B—C13B	5 (5)
C2—C7—C8—C60	−138.3 (11)	C3B—C4B—C12B—C13B	143 (3)
C36—C7—C8—C60	0.8 (18)	C5B—C4B—C12B—C11B	−141 (3)
C2—C7—C8—C9	0.6 (18)	C3B—C4B—C12B—C11B	−3 (3)
C36—C7—C8—C9	139.7 (12)	C11B—C12B—C13B—C17B	2 (3)
C7—C8—C9—C10	−0.5 (15)	C4B—C12B—C13B—C17B	−139 (3)
C60—C8—C9—C10	142.4 (9)	C11B—C12B—C13B—C14B	140 (2)
C7—C8—C9—C21	−142.5 (12)	C4B—C12B—C13B—C14B	−1 (4)
C60—C8—C9—C21	0.4 (12)	C12B—C13B—C14B—C51B	0 (4)
C21—C9—C10—C11	−1.4 (14)	C17B—C13B—C14B—C51B	140 (2)
C8—C9—C10—C11	−138.3 (8)	C12B—C13B—C14B—C15B	−143 (2)
C21—C9—C10—C3	137.6 (10)	C17B—C13B—C14B—C15B	−4 (3)
C8—C9—C10—C3	0.8 (12)	C51B—C14B—C15B—C40B	2 (4)
C2—C3—C10—C9	−1.2 (10)	C13B—C14B—C15B—C40B	144 (2)
C4—C3—C10—C9	−143.0 (8)	C51B—C14B—C15B—C16B	−139 (2)
C2—C3—C10—C11	142.1 (6)	C13B—C14B—C15B—C16B	3 (3)
C4—C3—C10—C11	0.3 (7)	C40B—C15B—C16B—C39B	2 (3)
C9—C10—C11—C19	0.5 (10)	C14B—C15B—C16B—C39B	145 (2)
C3—C10—C11—C19	−142.7 (6)	C40B—C15B—C16B—C17B	−144 (2)
C9—C10—C11—C12	142.4 (7)	C14B—C15B—C16B—C17B	−1 (3)
C3—C10—C11—C12	−0.7 (7)	C12B—C13B—C17B—C18B	−3 (4)
C5—C4—C12—C13	−0.5 (10)	C14B—C13B—C17B—C18B	−143 (3)
C3—C4—C12—C13	142.1 (6)	C12B—C13B—C17B—C16B	143 (2)
C5—C4—C12—C11	−143.3 (6)	C14B—C13B—C17B—C16B	3 (3)
C3—C4—C12—C11	−0.7 (7)	C39B—C16B—C17B—C18B	2 (4)
C19—C11—C12—C13	0.2 (9)	C15B—C16B—C17B—C18B	145 (2)
C10—C11—C12—C13	−142.8 (7)	C39B—C16B—C17B—C13B	−144 (3)
C19—C11—C12—C4	143.8 (7)	C15B—C16B—C17B—C13B	−1 (3)
C10—C11—C12—C4	0.9 (7)	C13B—C17B—C18B—C19B	4 (4)
C4—C12—C13—C17	−137.7 (8)	C16B—C17B—C18B—C19B	−137 (2)
C11—C12—C13—C17	0.5 (10)	C13B—C17B—C18B—C26B	140 (2)
C4—C12—C13—C14	0.7 (11)	C16B—C17B—C18B—C26B	−2 (4)
C11—C12—C13—C14	138.8 (7)	C12B—C11B—C19B—C18B	4 (3)
C12—C13—C14—C51	−1.0 (12)	C10B—C11B—C19B—C18B	141 (3)
C17—C13—C14—C51	141.0 (8)	C12B—C11B—C19B—C20B	−135 (2)
C12—C13—C14—C15	−143.9 (7)	C10B—C11B—C19B—C20B	2 (4)
C17—C13—C14—C15	−1.9 (9)	C17B—C18B—C19B—C11B	−5 (3)
C51—C14—C15—C40	−0.4 (12)	C26B—C18B—C19B—C11B	−144 (2)
C13—C14—C15—C40	142.9 (8)	C17B—C18B—C19B—C20B	137 (3)
C51—C14—C15—C16	−142.5 (9)	C26B—C18B—C19B—C20B	−2 (3)
C13—C14—C15—C16	0.8 (9)	C11B—C19B—C20B—C21B	1 (4)
C40—C15—C16—C39	0.3 (13)	C18B—C19B—C20B—C21B	−139 (3)
C14—C15—C16—C39	142.4 (9)	C11B—C19B—C20B—C25B	144 (2)
C40—C15—C16—C17	−141.6 (8)	C18B—C19B—C20B—C25B	4 (3)
C14—C15—C16—C17	0.5 (9)	C19B—C20B—C21B—C22B	133 (3)
C12—C13—C17—C18	−0.6 (11)	C25B—C20B—C21B—C22B	−4 (5)
C14—C13—C17—C18	−141.7 (7)	C19B—C20B—C21B—C9B	−4 (5)
C12—C13—C17—C16	143.3 (7)	C25B—C20B—C21B—C9B	−141 (3)
C14—C13—C17—C16	2.2 (9)	C10B—C9B—C21B—C20B	3 (6)
C39—C16—C17—C18	0.4 (13)	C8B—C9B—C21B—C20B	145 (4)
C15—C16—C17—C18	142.3 (7)	C10B—C9B—C21B—C22B	−140 (5)
C39—C16—C17—C13	−143.6 (10)	C8B—C9B—C21B—C22B	2 (5)
C15—C16—C17—C13	−1.7 (9)	C20B—C21B—C22B—C23B	8 (5)
C13—C17—C18—C19	0.1 (11)	C9B—C21B—C22B—C23B	145 (3)
C16—C17—C18—C19	−139.0 (8)	C20B—C21B—C22B—C60B	−140 (4)
C13—C17—C18—C26	139.4 (8)	C9B—C21B—C22B—C60B	−3 (5)
C16—C17—C18—C26	0.2 (11)	C60B—C22B—C23B—C31B	−6 (5)
C10—C11—C19—C18	137.6 (7)	C21B—C22B—C23B—C31B	−143 (3)
C12—C11—C19—C18	−0.6 (10)	C60B—C22B—C23B—C24B	129 (3)
C10—C11—C19—C20	−0.8 (10)	C21B—C22B—C23B—C24B	−8 (5)
C12—C11—C19—C20	−139.1 (7)	C22B—C23B—C24B—C25B	4 (4)
C17—C18—C19—C11	0.5 (11)	C31B—C23B—C24B—C25B	142 (3)
C26—C18—C19—C11	−142.3 (8)	C22B—C23B—C24B—C29B	−140 (3)
C17—C18—C19—C20	142.8 (7)	C31B—C23B—C24B—C29B	−2 (3)
C26—C18—C19—C20	0.1 (8)	C29B—C24B—C25B—C26B	1 (4)
C11—C19—C20—C21	2.2 (12)	C23B—C24B—C25B—C26B	−139 (2)
C18—C19—C20—C21	−140.6 (10)	C29B—C24B—C25B—C20B	140 (2)
C11—C19—C20—C25	142.2 (7)	C23B—C24B—C25B—C20B	0 (4)
C18—C19—C20—C25	−0.6 (8)	C21B—C20B—C25B—C24B	0 (4)
C19—C20—C21—C9	−3.1 (17)	C19B—C20B—C25B—C24B	−146 (2)
C25—C20—C21—C9	−139.5 (10)	C21B—C20B—C25B—C26B	142 (3)
C19—C20—C21—C22	137.1 (11)	C19B—C20B—C25B—C26B	−4 (3)
C25—C20—C21—C22	0.8 (17)	C24B—C25B—C26B—C27B	5 (4)
C10—C9—C21—C20	2.8 (18)	C20B—C25B—C26B—C27B	−136 (3)
C8—C9—C21—C20	144.5 (11)	C24B—C25B—C26B—C18B	144 (2)
C10—C9—C21—C22	−142.4 (9)	C20B—C25B—C26B—C18B	3 (3)
C8—C9—C21—C22	−0.6 (14)	C17B—C18B—C26B—C25B	−143 (2)
C20—C21—C22—C23	−0.4 (17)	C19B—C18B—C26B—C25B	−1 (2)
C9—C21—C22—C23	143.3 (9)	C17B—C18B—C26B—C27B	0 (4)
C20—C21—C22—C60	−143.1 (12)	C19B—C18B—C26B—C27B	142 (2)
C9—C21—C22—C60	0.6 (13)	C25B—C26B—C27B—C28B	−7 (4)
C60—C22—C23—C31	−1.2 (10)	C18B—C26B—C27B—C28B	−141 (2)
C21—C22—C23—C31	−138.6 (10)	C25B—C26B—C27B—C39B	136 (3)
C60—C22—C23—C24	137.8 (8)	C18B—C26B—C27B—C39B	2 (4)
C21—C22—C23—C24	0.4 (11)	C26B—C27B—C28B—C29B	3 (4)
C22—C23—C24—C25	−0.7 (10)	C39B—C27B—C28B—C29B	−145 (2)
C31—C23—C24—C25	142.7 (8)	C26B—C27B—C28B—C37B	148 (3)
C22—C23—C24—C29	−143.9 (6)	C39B—C27B—C28B—C37B	0 (3)
C31—C23—C24—C29	−0.5 (8)	C37B—C28B—C29B—C24B	−138 (2)
C29—C24—C25—C26	2.4 (11)	C27B—C28B—C29B—C24B	3 (3)
C23—C24—C25—C26	−136.9 (8)	C37B—C28B—C29B—C30B	−3 (4)
C29—C24—C25—C20	140.4 (7)	C27B—C28B—C29B—C30B	138 (3)
C23—C24—C25—C20	1.1 (11)	C25B—C24B—C29B—C28B	−5 (3)
C21—C20—C25—C24	−1.1 (12)	C23B—C24B—C29B—C28B	140 (2)
C19—C20—C25—C24	−142.2 (7)	C25B—C24B—C29B—C30B	−144 (3)
C21—C20—C25—C26	142.0 (10)	C23B—C24B—C29B—C30B	1 (3)
C19—C20—C25—C26	0.9 (9)	C28B—C29B—C30B—C35B	1 (6)
C24—C25—C26—C27	−0.1 (13)	C24B—C29B—C30B—C35B	142 (4)
C20—C25—C26—C27	−142.2 (9)	C28B—C29B—C30B—C31B	−141 (3)
C24—C25—C26—C18	141.3 (8)	C24B—C29B—C30B—C31B	1 (4)
C20—C25—C26—C18	−0.9 (9)	C22B—C23B—C31B—C32B	−1 (4)
C17—C18—C26—C27	−1.8 (13)	C24B—C23B—C31B—C32B	−143 (3)
C19—C18—C26—C27	141.8 (10)	C22B—C23B—C31B—C30B	145 (3)
C17—C18—C26—C25	−143.1 (7)	C24B—C23B—C31B—C30B	3 (4)
C19—C18—C26—C25	0.5 (9)	C35B—C30B—C31B—C32B	0 (6)
C25—C26—C27—C28	−1.5 (15)	C29B—C30B—C31B—C32B	143 (3)
C18—C26—C27—C28	−137.7 (10)	C35B—C30B—C31B—C23B	−145 (4)
C25—C26—C27—C39	138.9 (11)	C29B—C30B—C31B—C23B	−2 (4)
C18—C26—C27—C39	2.7 (16)	C23B—C31B—C32B—C33B	139 (3)
C26—C27—C28—C29	0.5 (15)	C30B—C31B—C32B—C33B	−2 (4)
C39—C27—C28—C29	−142.6 (9)	C23B—C31B—C32B—C57B	3 (4)
C26—C27—C28—C37	144.1 (10)	C30B—C31B—C32B—C57B	−138 (3)
C39—C27—C28—C37	1.0 (12)	C31B—C32B—C33B—C34B	3 (4)
C37—C28—C29—C30	0.6 (11)	C57B—C32B—C33B—C34B	147 (2)
C27—C28—C29—C30	138.2 (9)	C31B—C32B—C33B—C58B	−140 (2)
C37—C28—C29—C24	−135.8 (8)	C57B—C32B—C33B—C58B	5 (3)
C27—C28—C29—C24	1.9 (12)	C58B—C33B—C34B—C35B	138 (3)
C25—C24—C29—C28	−3.4 (11)	C32B—C33B—C34B—C35B	−2 (3)
C23—C24—C29—C28	141.3 (7)	C58B—C33B—C34B—C44B	−1 (3)
C25—C24—C29—C30	−143.7 (7)	C32B—C33B—C34B—C44B	−141 (2)
C23—C24—C29—C30	1.1 (8)	C31B—C30B—C35B—C34B	1 (6)
C28—C29—C30—C35	0.2 (10)	C29B—C30B—C35B—C34B	−137 (3)
C24—C29—C30—C35	141.9 (7)	C31B—C30B—C35B—C42B	138 (3)
C28—C29—C30—C31	−143.0 (7)	C29B—C30B—C35B—C42B	0 (6)
C24—C29—C30—C31	−1.3 (8)	C33B—C34B—C35B—C30B	0 (5)
C22—C23—C31—C32	0.9 (12)	C44B—C34B—C35B—C30B	142 (4)
C24—C23—C31—C32	−142.3 (8)	C33B—C34B—C35B—C42B	−143 (3)
C22—C23—C31—C30	142.8 (7)	C44B—C34B—C35B—C42B	0 (3)
C24—C23—C31—C30	−0.3 (9)	C8B—C7B—C36B—C56B	−6 (4)
C35—C30—C31—C32	−1.0 (12)	C2B—C7B—C36B—C56B	136 (3)
C29—C30—C31—C32	142.6 (8)	C8B—C7B—C36B—C55B	−142 (3)
C35—C30—C31—C23	−142.6 (7)	C2B—C7B—C36B—C55B	0 (3)
C29—C30—C31—C23	1.0 (9)	C29B—C28B—C37B—C42B	4 (4)
C23—C31—C32—C33	137.9 (8)	C27B—C28B—C37B—C42B	−140 (3)
C30—C31—C32—C33	0.7 (13)	C29B—C28B—C37B—C38B	143 (2)
C23—C31—C32—C57	−1.1 (13)	C27B—C28B—C37B—C38B	−2 (3)
C30—C31—C32—C57	−138.3 (8)	C42B—C37B—C38B—C41B	2 (4)
C31—C32—C33—C58	−141.6 (9)	C28B—C37B—C38B—C41B	−140 (3)
C57—C32—C33—C58	2.5 (9)	C42B—C37B—C38B—C39B	144 (3)
C31—C32—C33—C34	0.7 (12)	C28B—C37B—C38B—C39B	3 (3)
C57—C32—C33—C34	144.8 (7)	C15B—C16B—C39B—C27B	−140 (3)
C58—C33—C34—C35	136.4 (8)	C17B—C16B—C39B—C27B	−1 (4)
C32—C33—C34—C35	−1.9 (10)	C15B—C16B—C39B—C38B	−9 (4)
C58—C33—C34—C44	−0.7 (10)	C17B—C16B—C39B—C38B	130 (3)
C32—C33—C34—C44	−139.0 (7)	C28B—C27B—C39B—C16B	143 (3)
C29—C30—C35—C34	−139.4 (7)	C26B—C27B—C39B—C16B	−1 (5)
C31—C30—C35—C34	−0.1 (10)	C28B—C27B—C39B—C38B	1 (3)
C29—C30—C35—C42	−0.9 (11)	C26B—C27B—C39B—C38B	−143 (3)
C31—C30—C35—C42	138.3 (8)	C41B—C38B—C39B—C16B	8 (4)
C33—C34—C35—C30	1.7 (10)	C37B—C38B—C39B—C16B	−138 (3)
C44—C34—C35—C30	142.7 (6)	C41B—C38B—C39B—C27B	144 (3)
C33—C34—C35—C42	−142.2 (6)	C37B—C38B—C39B—C27B	−2 (3)
C44—C34—C35—C42	−1.3 (7)	C16B—C15B—C40B—C49B	141 (2)
C8—C7—C36—C56	−2.5 (19)	C14B—C15B—C40B—C49B	2 (3)
C2—C7—C36—C56	141.1 (10)	C16B—C15B—C40B—C41B	5 (4)
C8—C7—C36—C55	−144.2 (12)	C14B—C15B—C40B—C41B	−134 (3)
C2—C7—C36—C55	−0.6 (14)	C37B—C38B—C41B—C59B	−1 (5)
C29—C28—C37—C42	−0.6 (11)	C39B—C38B—C41B—C59B	−141 (3)
C27—C28—C37—C42	−143.0 (8)	C37B—C38B—C41B—C40B	139 (3)
C29—C28—C37—C38	141.5 (10)	C39B—C38B—C41B—C40B	−1 (4)
C27—C28—C37—C38	−0.9 (11)	C15B—C40B—C41B—C38B	−5 (4)
C42—C37—C38—C41	3.0 (17)	C49B—C40B—C41B—C38B	−143 (3)
C28—C37—C38—C41	−140.8 (11)	C15B—C40B—C41B—C59B	137 (3)
C42—C37—C38—C39	144.3 (9)	C49B—C40B—C41B—C59B	−1 (4)
C28—C37—C38—C39	0.5 (13)	C28B—C37B—C42B—C43B	134 (4)
C15—C16—C39—C27	−136.9 (11)	C38B—C37B—C42B—C43B	−2 (5)
C17—C16—C39—C27	0.5 (17)	C28B—C37B—C42B—C35B	−4 (4)
C15—C16—C39—C38	−1.1 (16)	C38B—C37B—C42B—C35B	−139 (3)
C17—C16—C39—C38	136.3 (11)	C30B—C35B—C42B—C37B	2 (5)
C26—C27—C39—C16	−2.1 (18)	C34B—C35B—C42B—C37B	144 (3)
C28—C27—C39—C16	141.1 (11)	C30B—C35B—C42B—C43B	−140 (4)
C26—C27—C39—C38	−143.9 (12)	C34B—C35B—C42B—C43B	2 (4)
C28—C27—C39—C38	−0.7 (14)	C37B—C42B—C43B—C59B	1 (7)
C41—C38—C39—C16	1.1 (19)	C35B—C42B—C43B—C59B	141 (5)
C37—C38—C39—C16	−142.1 (11)	C37B—C42B—C43B—C44B	−143 (3)
C41—C38—C39—C27	143.3 (12)	C35B—C42B—C43B—C44B	−3 (5)
C37—C38—C39—C27	0.1 (14)	C59B—C43B—C44B—C45B	3 (6)
C16—C15—C40—C49	139.3 (8)	C42B—C43B—C44B—C45B	147 (3)
C14—C15—C40—C49	1.4 (12)	C59B—C43B—C44B—C34B	−142 (4)
C16—C15—C40—C41	0.5 (13)	C42B—C43B—C44B—C34B	3 (4)
C14—C15—C40—C41	−137.4 (9)	C33B—C34B—C44B—C45B	1 (3)
C37—C38—C41—C40	136.8 (11)	C35B—C34B—C44B—C45B	−144 (2)
C39—C38—C41—C40	−0.4 (17)	C33B—C34B—C44B—C43B	143 (3)
C37—C38—C41—C59	−1.0 (18)	C35B—C34B—C44B—C43B	−2 (4)
C39—C38—C41—C59	−138.2 (11)	C43B—C44B—C45B—C46B	−140 (3)
C15—C40—C41—C38	−0.4 (14)	C34B—C44B—C45B—C46B	0 (3)
C49—C40—C41—C38	−143.2 (10)	C43B—C44B—C45B—C47B	−5 (4)
C15—C40—C41—C59	141.2 (9)	C34B—C44B—C45B—C47B	135 (2)
C49—C40—C41—C59	−1.6 (10)	C44B—C45B—C46B—C58B	0 (3)
C28—C37—C42—C43	137.6 (7)	C47B—C45B—C46B—C58B	−138 (2)
C38—C37—C42—C43	−2.6 (11)	C44B—C45B—C46B—C54B	142.4 (19)
C28—C37—C42—C35	−0.2 (10)	C47B—C45B—C46B—C54B	4 (2)
C38—C37—C42—C35	−140.4 (9)	C44B—C45B—C47B—C48B	2 (4)
C30—C35—C42—C37	1.0 (10)	C46B—C45B—C47B—C48B	139 (3)
C34—C35—C42—C37	143.9 (6)	C44B—C45B—C47B—C53B	−143 (2)
C30—C35—C42—C43	−141.4 (7)	C46B—C45B—C47B—C53B	−5 (3)
C34—C35—C42—C43	1.5 (7)	C53B—C47B—C48B—C59B	143 (3)
C37—C42—C43—C59	0.3 (10)	C45B—C47B—C48B—C59B	3 (5)
C35—C42—C43—C59	140.7 (7)	C53B—C47B—C48B—C49B	8 (5)
C37—C42—C43—C44	−141.5 (6)	C45B—C47B—C48B—C49B	−132 (3)
C35—C42—C43—C44	−1.2 (7)	C15B—C40B—C49B—C50B	−6 (4)
C59—C43—C44—C45	1.2 (10)	C41B—C40B—C49B—C50B	139 (3)
C42—C43—C44—C45	144.0 (6)	C15B—C40B—C49B—C48B	−148 (3)
C59—C43—C44—C34	−142.5 (7)	C41B—C40B—C49B—C48B	−3 (3)
C42—C43—C44—C34	0.4 (7)	C47B—C48B—C49B—C50B	−9 (5)
C33—C34—C44—C45	−0.1 (9)	C59B—C48B—C49B—C50B	−143 (3)
C35—C34—C44—C45	−143.2 (7)	C47B—C48B—C49B—C40B	139 (3)
C33—C34—C44—C43	143.6 (6)	C59B—C48B—C49B—C40B	6 (4)
C35—C34—C44—C43	0.6 (7)	C40B—C49B—C50B—C51B	5 (4)
C43—C44—C45—C46	−138.6 (7)	C48B—C49B—C50B—C51B	145 (3)
C34—C44—C45—C46	1.3 (9)	C40B—C49B—C50B—C52B	−133 (3)
C43—C44—C45—C47	−1.9 (9)	C48B—C49B—C50B—C52B	6 (4)
C34—C44—C45—C47	138.0 (7)	C13B—C14B—C51B—C5B	−2 (4)
C44—C45—C46—C58	−1.5 (10)	C15B—C14B—C51B—C5B	134 (3)
C47—C45—C46—C58	−143.1 (7)	C13B—C14B—C51B—C50B	−139 (2)
C44—C45—C46—C54	141.4 (7)	C15B—C14B—C51B—C50B	−3 (3)
C47—C45—C46—C54	−0.1 (8)	C4B—C5B—C51B—C14B	6 (6)
C44—C45—C47—C48	1.1 (11)	C6B—C5B—C51B—C14B	−144 (3)
C46—C45—C47—C48	142.6 (9)	C4B—C5B—C51B—C50B	144 (4)
C44—C45—C47—C53	−141.4 (6)	C6B—C5B—C51B—C50B	−5 (4)
C46—C45—C47—C53	0.0 (8)	C49B—C50B—C51B—C14B	0 (3)
C53—C47—C48—C59	139.0 (10)	C52B—C50B—C51B—C14B	144 (2)
C45—C47—C48—C59	0.5 (16)	C49B—C50B—C51B—C5B	−141 (3)
C53—C47—C48—C49	1.7 (16)	C52B—C50B—C51B—C5B	3 (3)
C45—C47—C48—C49	−136.8 (9)	C49B—C50B—C52B—C53B	−2 (4)
C15—C40—C49—C50	−1.5 (12)	C51B—C50B—C52B—C53B	−144 (2)
C41—C40—C49—C50	143.1 (7)	C49B—C50B—C52B—C6B	142 (2)
C15—C40—C49—C48	−143.9 (10)	C51B—C50B—C52B—C6B	0 (2)
C41—C40—C49—C48	0.8 (10)	C1B—C6B—C52B—C53B	4 (3)
C47—C48—C49—C50	−2.5 (15)	C5B—C6B—C52B—C53B	142 (3)
C59—C48—C49—C50	−143.8 (8)	C1B—C6B—C52B—C50B	−141.4 (19)
C47—C48—C49—C40	141.7 (11)	C5B—C6B—C52B—C50B	−3 (3)
C59—C48—C49—C40	0.4 (12)	C50B—C52B—C53B—C54B	140 (2)
C40—C49—C50—C51	0.6 (10)	C6B—C52B—C53B—C54B	0 (3)
C48—C49—C50—C51	139.8 (9)	C50B—C52B—C53B—C47B	0 (3)
C40—C49—C50—C52	−138.0 (8)	C6B—C52B—C53B—C47B	−139 (2)
C48—C49—C50—C52	1.2 (10)	C48B—C47B—C53B—C52B	−4 (4)
C13—C14—C51—C5	1.0 (13)	C45B—C47B—C53B—C52B	145 (2)
C15—C14—C51—C5	138.7 (8)	C48B—C47B—C53B—C54B	−144 (3)
C13—C14—C51—C50	−138.3 (8)	C45B—C47B—C53B—C54B	5 (3)
C15—C14—C51—C50	−0.5 (12)	C52B—C53B—C54B—C55B	−5 (4)
C4—C5—C51—C14	−0.8 (11)	C47B—C53B—C54B—C55B	136 (3)
C6—C5—C51—C14	−143.3 (8)	C52B—C53B—C54B—C46B	−143 (2)
C4—C5—C51—C50	141.6 (7)	C47B—C53B—C54B—C46B	−2 (3)
C6—C5—C51—C50	−0.9 (9)	C58B—C46B—C54B—C55B	0 (3)
C49—C50—C51—C14	0.4 (11)	C45B—C46B—C54B—C55B	−144 (2)
C52—C50—C51—C14	143.7 (8)	C58B—C46B—C54B—C53B	143 (2)
C49—C50—C51—C5	−142.2 (7)	C45B—C46B—C54B—C53B	−1 (2)
C52—C50—C51—C5	1.0 (9)	C53B—C54B—C55B—C1B	5 (4)
C49—C50—C52—C53	0.9 (9)	C46B—C54B—C55B—C1B	139 (2)
C51—C50—C52—C53	−142.4 (7)	C53B—C54B—C55B—C36B	−135 (3)
C49—C50—C52—C6	142.6 (6)	C46B—C54B—C55B—C36B	−1 (4)
C51—C50—C52—C6	−0.7 (7)	C2B—C1B—C55B—C54B	−144 (3)
C1—C6—C52—C53	1.3 (9)	C6B—C1B—C55B—C54B	−1 (3)
C5—C6—C52—C53	143.7 (6)	C2B—C1B—C55B—C36B	2 (3)
C1—C6—C52—C50	−142.2 (6)	C6B—C1B—C55B—C36B	145 (2)
C5—C6—C52—C50	0.2 (7)	C56B—C36B—C55B—C54B	1 (4)
C50—C52—C53—C54	136.7 (8)	C7B—C36B—C55B—C54B	142 (3)
C6—C52—C53—C54	−0.7 (10)	C56B—C36B—C55B—C1B	−142 (3)
C50—C52—C53—C47	−1.7 (10)	C7B—C36B—C55B—C1B	−1 (3)
C6—C52—C53—C47	−139.1 (7)	C55B—C36B—C56B—C57B	139 (3)
C48—C47—C53—C52	0.4 (11)	C7B—C36B—C56B—C57B	8 (4)
C45—C47—C53—C52	143.1 (6)	C55B—C36B—C56B—C58B	−1 (4)
C48—C47—C53—C54	−142.6 (10)	C7B—C36B—C56B—C58B	−132 (3)
C45—C47—C53—C54	0.1 (8)	C36B—C56B—C57B—C60B	−3 (3)
C52—C53—C54—C55	−0.7 (13)	C58B—C56B—C57B—C60B	143 (2)
C47—C53—C54—C55	140.9 (9)	C36B—C56B—C57B—C32B	−143 (2)
C52—C53—C54—C46	−141.8 (7)	C58B—C56B—C57B—C32B	3 (3)
C47—C53—C54—C46	−0.2 (8)	C31B—C32B—C57B—C60B	0 (4)
C58—C46—C54—C55	0.9 (12)	C33B—C32B—C57B—C60B	−144 (2)
C45—C46—C54—C55	−142.1 (9)	C31B—C32B—C57B—C56B	139 (3)
C58—C46—C54—C53	143.2 (7)	C33B—C32B—C57B—C56B	−4 (3)
C45—C46—C54—C53	0.2 (8)	C45B—C46B—C58B—C33B	0 (3)
C53—C54—C55—C1	1.5 (15)	C54B—C46B—C58B—C33B	−138 (2)
C46—C54—C55—C1	138.1 (9)	C45B—C46B—C58B—C56B	139 (2)
C53—C54—C55—C36	−138.4 (10)	C54B—C46B—C58B—C56B	1 (3)
C46—C54—C55—C36	−1.8 (15)	C34B—C33B—C58B—C46B	0 (3)
C6—C1—C55—C54	−0.9 (14)	C32B—C33B—C58B—C46B	141 (2)
C2—C1—C55—C54	−144.2 (10)	C34B—C33B—C58B—C56B	−144 (2)
C6—C1—C55—C36	142.2 (9)	C32B—C33B—C58B—C56B	−3 (3)
C2—C1—C55—C36	−1.1 (11)	C36B—C56B—C58B—C46B	0 (4)
C56—C36—C55—C54	2.1 (17)	C57B—C56B—C58B—C46B	−144 (2)
C7—C36—C55—C54	143.9 (12)	C36B—C56B—C58B—C33B	144 (3)
C56—C36—C55—C1	−140.7 (11)	C57B—C56B—C58B—C33B	0 (3)
C7—C36—C55—C1	1.0 (13)	C42B—C43B—C59B—C41B	0 (7)
C55—C36—C56—C57	137.7 (11)	C44B—C43B—C59B—C41B	139 (4)
C7—C36—C56—C57	2.6 (16)	C42B—C43B—C59B—C48B	−135 (4)
C55—C36—C56—C58	−1.4 (16)	C44B—C43B—C59B—C48B	3 (8)
C7—C36—C56—C58	−136.5 (12)	C38B—C41B—C59B—C43B	0 (6)
C36—C56—C57—C60	−1.0 (13)	C40B—C41B—C59B—C43B	−141 (4)
C58—C56—C57—C60	142.9 (8)	C38B—C41B—C59B—C48B	146 (3)
C36—C56—C57—C32	−142.9 (9)	C40B—C41B—C59B—C48B	4 (4)
C58—C56—C57—C32	1.0 (9)	C47B—C48B—C59B—C43B	−6 (6)
C31—C32—C57—C60	1.8 (12)	C49B—C48B—C59B—C43B	134 (5)
C33—C32—C57—C60	−143.3 (8)	C47B—C48B—C59B—C41B	−147 (3)
C31—C32—C57—C56	143.0 (9)	C49B—C48B—C59B—C41B	−6 (4)
C33—C32—C57—C56	−2.1 (9)	C23B—C22B—C60B—C57B	9 (5)
C45—C46—C58—C33	0.7 (10)	C21B—C22B—C60B—C57B	148 (3)
C54—C46—C58—C33	−138.0 (8)	C23B—C22B—C60B—C8B	−137 (3)
C45—C46—C58—C56	138.5 (8)	C21B—C22B—C60B—C8B	3 (4)
C54—C46—C58—C56	−0.2 (11)	C56B—C57B—C60B—C22B	−143 (3)
C34—C33—C58—C46	0.5 (10)	C32B—C57B—C60B—C22B	−5 (3)
C32—C33—C58—C46	140.4 (7)	C56B—C57B—C60B—C8B	−3 (4)
C34—C33—C58—C56	−141.8 (7)	C32B—C57B—C60B—C8B	135 (3)
C32—C33—C58—C56	−1.9 (9)	C7B—C8B—C60B—C22B	146 (3)
C36—C56—C58—C46	0.4 (13)	C9B—C8B—C60B—C22B	−1 (4)
C57—C56—C58—C46	−142.8 (8)	C7B—C8B—C60B—C57B	4 (5)
C36—C56—C58—C33	143.7 (10)	C9B—C8B—C60B—C57B	−143 (3)
C57—C56—C58—C33	0.5 (9)	C68—C61—C62—C69	2.7 (6)
C44—C43—C59—C48	0.5 (13)	C66—C61—C62—C69	−178.0 (4)
C42—C43—C59—C48	−137.0 (10)	C68—C61—C62—C63	−176.7 (3)
C44—C43—C59—C41	139.3 (8)	C66—C61—C62—C63	2.6 (5)
C42—C43—C59—C41	1.8 (12)	C69—C62—C63—C70	−1.2 (5)
C47—C48—C59—C43	−1.3 (18)	C61—C62—C63—C70	178.3 (3)
C49—C48—C59—C43	141.6 (9)	C69—C62—C63—C64	178.1 (4)
C47—C48—C59—C41	−144.2 (11)	C61—C62—C63—C64	−2.4 (5)
C49—C48—C59—C41	−1.4 (13)	C70—C63—C64—C71	−0.2 (7)
C38—C41—C59—C43	−1.5 (14)	C62—C63—C64—C71	−179.3 (4)
C40—C41—C59—C43	−142.7 (8)	C70—C63—C64—C65	179.3 (4)
C38—C41—C59—C48	143.1 (12)	C62—C63—C64—C65	0.2 (5)
C40—C41—C59—C48	1.9 (11)	C71—C64—C65—C72	0.2 (5)
C56—C57—C60—C8	−0.7 (12)	C63—C64—C65—C72	−179.3 (4)
C32—C57—C60—C8	136.4 (9)	C71—C64—C65—C66	−178.5 (3)
C56—C57—C60—C22	−139.2 (8)	C63—C64—C65—C66	1.9 (5)
C32—C57—C60—C22	−2.1 (12)	C68—C61—C66—C67	0.1 (5)
C7—C8—C60—C57	0.7 (15)	C62—C61—C66—C67	−179.3 (3)
C9—C8—C60—C57	−142.0 (9)	C68—C61—C66—C65	178.9 (3)
C7—C8—C60—C22	142.7 (10)	C62—C61—C66—C65	−0.5 (5)
C9—C8—C60—C22	0.0 (10)	C72—C65—C66—C67	−1.7 (6)
C23—C22—C60—C57	1.9 (11)	C64—C65—C66—C67	176.8 (3)
C21—C22—C60—C57	143.3 (10)	C72—C65—C66—C61	179.7 (4)
C23—C22—C60—C8	−141.8 (7)	C64—C65—C66—C61	−1.8 (5)
C21—C22—C60—C8	−0.4 (11)	C61—C66—C67—S1	−0.2 (4)
C6B—C1B—C2B—C3B	−3 (4)	C65—C66—C67—S1	−178.8 (3)
C55B—C1B—C2B—C3B	139 (3)	C68—S1—C67—C66	0.1 (3)
C6B—C1B—C2B—C7B	−144 (2)	C66—C61—C68—S1	0.0 (4)
C55B—C1B—C2B—C7B	−2 (3)	C62—C61—C68—S1	179.4 (3)
C1B—C2B—C3B—C10B	−133 (4)	C67—S1—C68—C61	−0.1 (3)
C7B—C2B—C3B—C10B	3 (5)	C63—C62—C69—S2	1.0 (4)
C1B—C2B—C3B—C4B	5 (5)	C61—C62—C69—S2	−178.5 (3)
C7B—C2B—C3B—C4B	141 (3)	C70—S2—C69—C62	−0.4 (3)
C2B—C3B—C4B—C5B	−4 (5)	C62—C63—C70—S2	0.9 (5)
C10B—C3B—C4B—C5B	139 (4)	C64—C63—C70—S2	−178.3 (3)
C2B—C3B—C4B—C12B	−142 (3)	C69—S2—C70—C63	−0.3 (4)
C10B—C3B—C4B—C12B	1 (4)	C65—C64—C71—S3	0.3 (4)
C12B—C4B—C5B—C51B	−7 (6)	C63—C64—C71—S3	179.8 (3)
C3B—C4B—C5B—C51B	−141 (4)	C72—S3—C71—C64	−0.5 (4)
C12B—C4B—C5B—C6B	137 (4)	C64—C65—C72—S3	−0.7 (4)
C3B—C4B—C5B—C6B	3 (6)	C66—C65—C72—S3	177.9 (3)
C2B—C1B—C6B—C5B	1 (3)	C71—S3—C72—C65	0.7 (3)
C55B—C1B—C6B—C5B	−138 (3)	C78—C73—C74—C75	−0.1 (7)
C2B—C1B—C6B—C52B	137 (2)	C73—C74—C75—C76	−0.2 (7)
C55B—C1B—C6B—C52B	−3 (3)	C74—C75—C76—C77	0.3 (7)
C4B—C5B—C6B—C1B	−1 (6)	C75—C76—C77—C78	−0.1 (7)
C51B—C5B—C6B—C1B	146 (3)	C76—C77—C78—C73	−0.2 (6)
C4B—C5B—C6B—C52B	−142 (4)	C76—C77—C78—C79	−178.9 (4)
C51B—C5B—C6B—C52B	5 (4)	C74—C73—C78—C77	0.3 (6)
C3B—C2B—C7B—C8B	−4 (4)	C74—C73—C78—C79	179.0 (4)
